# Emotional literacy and levels of consciousness in bio-psycho-social-spiritual-ecological (5D) model of human experience

**DOI:** 10.3389/fpsyg.2026.1520959

**Published:** 2026-01-22

**Authors:** Ivana Dragic

**Affiliations:** Faculty of Psychotherapy Science, Sigmund Freud Private University, Vienna, Austria

**Keywords:** emotional literacy, emotion regulation, the circular emotional reaction model, levels of consciousness, bio-psycho-social-spiritual-ecological model, 5D model

## Abstract

The efficacy of emotion regulation, critical for psychological wellbeing and a transdiagnostic approach to treating mental health, is tightly related to a broader emotional literacy construct. Both concepts are inseparably intertwined with a conceptual framework of emotions, but a generally accepted theory is still missing. This article presents the circular emotional reaction (CER) model by Zoran Milivojevic as a comprehensive framework of emotion psychology and psychopathology that aligns with the latest scientific findings. It further elaborates on the framework by introducing the novel bio-psycho-social-spiritual-ecological (5D) model of human experience, depicted as an iceberg personality metaphor that integrates major psychological functions through the lifespan on the intrapersonal, interpersonal, and societal level, with the CER model as a link between the different domains. Against that backdrop, distinct levels of consciousness are formulated that are informed by moral development and the locus of power, and the main hypothesis is postulated that they are directly proportional to emotional literacy. Despite its complexity, the developed model offers practical tools by laying out the continuum of the structural inadequacy of emotional reactions, serving as the foundation for diagnosing and treating mental health disorders and developing emotion regulation skills crucial for psychological wellbeing and conscious evolution.

## 1 Introduction

Emotion regulation has recently been recognized as critical for psychological wellbeing and as a transdiagnostic factor in the treatment of mental health disorders (Kring and Sloan, 2009; Sheppes et al., 2015; Beauchaine and Cicchetti, 2019). The field of emotion regulation emerged in the mid-1990s and commonly relies on the model developed by Gross (1999) and subsequently his extended process model of emotion regulation (Gross, 2015). However, without emotional literacy, emotion regulation alone can be misunderstood and inadvertently used to preserve the unhealthy status quo—for example, by withholding anger in a situation where it should be more adequate to express it assertively and prevent injustice. Steiner (2003), who coined the term emotional literacy, described it as an ability “to handle emotions in a way that improves your power…and improves the quality of life of the people around you” (p.1). Both concepts are inseparably intertwined with a conceptual framework of emotions, though a generally accepted theory is still missing.

Gendron and Feldman Barrett (2009) reconstructed the history of the scientific study of emotion in psychology, and they outlined three fundamental approaches: (1) evolutionary “basic emotion” approach that emphasizes the adaptive functions of emotions (e.g., James, 1884; Tooby and Cosmides, 1990); (2) “appraisal” approach, which assumes that emotions arise from interpretation (e.g., Arnold, 1960; Frijda, 1986); and (3) the more recent “psychological constructionist” approach (e.g., Russell, 2003; Barrett, 2006). Reisenzein (2015) reviewed the history of psychological theory and research on emotion according to five main questions: “(1) the causal generation of emotions, (2) the effects of emotion on subsequent cognition and behavior, (3) the nature of emotion, (4) the evolutionary and learning origins of the emotion system, and (5) the neural structures and processes involved in emotions” (p.21). He concluded that a “generally accepted theory of emotions that gives detailed answers to all these questions, or even just to the central questions Q1-Q3, still does not exist today” (p. 24).

After introducing Steiner’s (2003) concept of emotional literacy, this article presents Zoran Milivojevic’s (2000) circular emotional reaction (CER) model as a comprehensive framework of emotion that unifies evolutionary, appraisal, and constructionist approaches in line with the latest scientific findings. It then introduces the novel bio-psycho-social-spiritual-ecological (5D) model of human experience, depicted as an iceberg personality metaphor that integrates major psychological functions across the lifespan at intrapersonal, interpersonal, and societal levels, with the CER model linking its domains. Against that backdrop, distinct levels of consciousness are formulated, informed by moral development and the locus of power, and the main hypothesis is postulated: they are directly proportional to emotional literacy. This hypothesis is further extended to include other psychological functions involved in the personal iceberg—making the main assumption, despite its elusive concepts, empirically testable. Taken together, the developed 5D model offers a theoretical framework on emotion that addresses all five core questions of emotion psychology raised by Reisenzein (2015) and invites both research testing of the proposed hypotheses and theoretical dialogue. Possible applications for emotion regulation are briefly outlined, implications for the proposed research are indicated, and broader implications are noted, within the constraints of a single article.

## 2 Emotional literacy

The term “emotional intelligence” is more widely known than “emotional literacy,” gaining popularity especially after Goleman’s (1995) book of the same name. Originally, Salovey and Mayer (1990) defined emotional intelligence as “the ability to monitor one’s own and others’ feelings and emotions, to discriminate among them, and use this information to guide one’s thinking and actions.” This concept has since inspired extensive research and development. However, without a deep understanding of emotion, emotional intelligence can be unintentionally—or intentionally—used for manipulation and subtle forms of power abuse. Emotional literacy aims to enhance the effectiveness and growth of emotional intelligence. The term was coined by Steiner (1979) in his book *Healing Alcoholism*. He defined it as “*love-centered emotional intelligence*. Loving (oneself and others) and being loved (by oneself and others) are the essential conditions of emotional literacy” (Steiner, 2003, p. 11).

**Figure 1:**
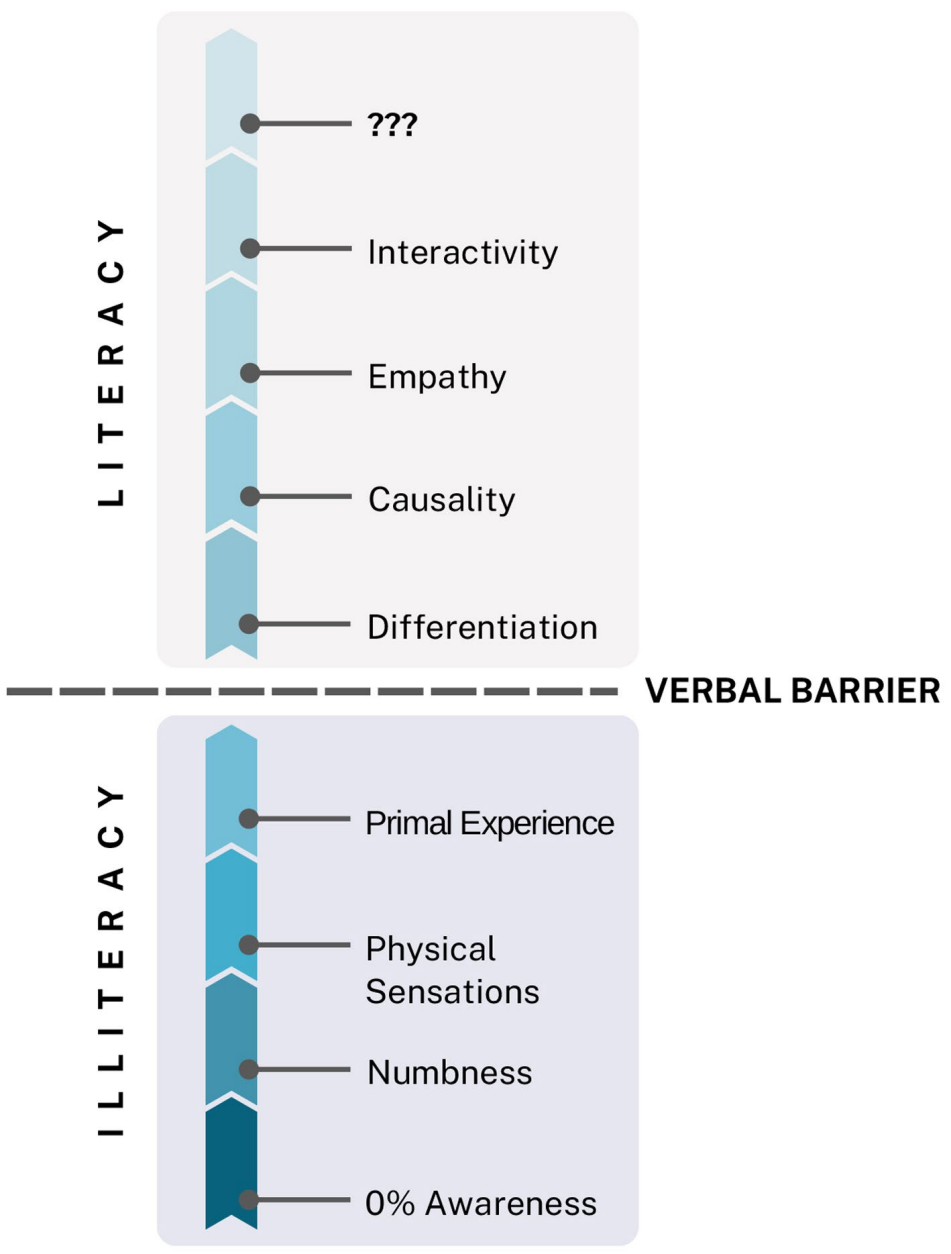
The Emotional Awareness Scale. The scale begins at a symbolic zero percent to indicate the upward progression of awareness. The levels move from numbness (detachment—markedly reduced or absent subjective feeling), to physical sensations (somatization—bodily signs such as palpitations without linking them to emotions), to chaotic primal experience (intense feelings without understanding or words). Crossing the verbal barrier (the first steps in naming emotions, supported by an emotionally open environment) marks the start of conscious articulation. Differentiation (enabling recognition, verbalization, and a growing ability to hold multiple emotions at once) follows, and then causality (identifying triggers and causes of feelings). Empathy (understanding a person from their frame of reference and discerning their emotions) emerges as a higher-order competency, leading to interactivity (skilled navigation of emotional exchanges, with anticipation of their escalation or resolution). Above this, Steiner placed question marks (an intentionally open space for potential higher levels of emotional literacy, inviting further conceptual development). Adapted from Steiner (2003).

Figure 1 illustrates the emotional awareness scale, a core aspect of emotional literacy. Lane and Smith (2021) developed the Levels of Emotional Awareness Scale (LEAS), a reliable and valid tool based on concepts similar to Steiner’s. Their meta-review of LEAS studies shows that emotional awareness promotes better self-regulation, improved handling of complex social situations, stronger relationships, and enhanced physical and mental health. Steiner (2003) identified interactivity—proficiency in “speaking the language of emotions”—as the crucial link between emotional awareness and emotional literacy. Emotional literacy can be developed through training that increases awareness of emotions in ourselves and others, fosters honesty, encourages responsibility for our actions, and reconnects us with the power of feelings, especially the power of love. This central idea will be explored throughout the article.

## 3 The circular emotional reaction model

The CER model by Zoran Milivojevic (2000) is a comprehensive framework of emotion psychology and psychopathology, first presented in 1993 in his book *Emotions: Psychotherapy and Understanding of Emotions* (*Emocije: Psihoterapija i Razumevanje Emocija*; author’s translation). An overview of the CER model as an explication of the body–mind connection can be found in Dragic (2021). Here, the main elements of the CER model as an emotion-psychology framework will be outlined and supported by relevant neuroscientific developments, followed by a brief discussion of how the model understands emotional awareness, and an outline of the continuum of structural inadequacy in emotional reactions as a basis for psychopathology.

### 3.1 The main elements of the CER model

**Figure 2:**
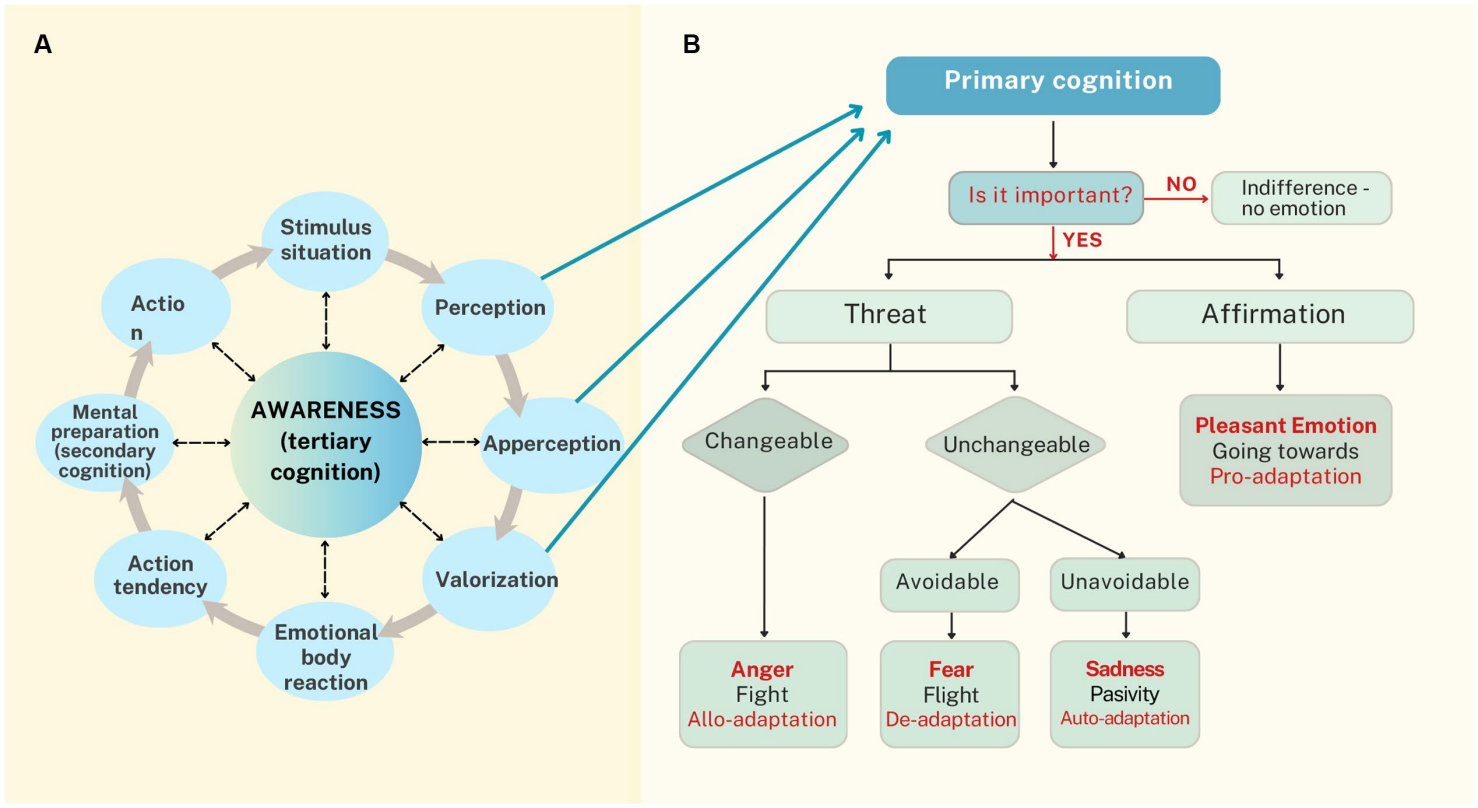
**(A)** The Circular Emotional Reaction (CER) Model. Perception, apperception, and valorization serve as features of our *primary cognition*, which continually uses a frame of reference to assess a stimulus situation and activate specific adaptive reactions in case of an important change. These will initiate respective emotional body reactions and lead to an expression of emotion that includes action tendency, mental preparation as a *secondarycognition*, and action toward the stimulus situation. We can be unaware of all or of some steps in a sequence and to different degrees, but awareness as a *tertiary cognition* in the CER model is a precondition for conscious emotion regulation. **(B)** Primary cognition and the choice of adaptive emotional reaction. When primary cognition detects a significant change in circumstances, it initiates adaptive emotional reactions by assessing whether the situation affirms or threatens one’s values, thereby triggering pleasant or unpleasant emotions and preparing the body for action. Unpleasant emotions drive allo-adaptation (changing circumstances), de-adaptation (avoiding them), or auto-adaptation (mentally adjusting tounchangeable realities). Pleasant emotions prompt behaviors that sustain or enhance affirming situations (pro-adaptation), combining environmental shaping with the integration of positive changes into one’s frame of reference. Adapted from Milivojevic (2000).

Milivojevic (2000) defined emotion as “a reaction of a subject to a stimulus situation, which was valued as important, and which prepares a subject for adaptive behavior on visceral, motoric, motivational, and mental levels” (p. 15). Emotions arise when an individual *appraises a significant change* in the relationship between self and environment relative to their frame of reference, aligning with appraisal theories of emotion. These reactions both result from such changes and aim to restore balance, reflecting *the adaptive function* emphasized in evolutionary approaches to emotion. This definition highlights the reactive nature of emotions as qualitatively personal responses to lived situations, inseparable from the relational context between organism and environment. Milivojevic further argued that the term *emotional reaction* is more precise, referring to a complex, *circular mechanism integrating cognitive, physiological, and behavioral components in eight sequential steps*: stimulus situation, perception, apperception, valorization, emotional body reaction, action tendency, mental preparation, and action affecting the stimulus situation (see Figure 2A). The CER model posits that these steps reflect basic psychological functions operating in both emotional and non-emotional states. This view aligns with meta-analytic findings in affective neuroscience (Lindquist et al., 2012) showing that emotion experience and perception engage interacting brain networks involved in fundamental psychological operations, rather than discrete, localized emotion centers. It also resonates with constructionist approaches to emotion, such as those advanced by Gendron and Feldman Barrett (2009).

Distinctions between three levels of cognition in the CER model, as shown in Figure 2A, correspond with the essential neuroevolutionary view postulated by MacLean (1990), who conceptualized the evolution of the triune human brain with overlapping layers: (1) *the “reptilian” brain* that refers to subcortical midline structures as they are already present in reptiles; (2) *the intermediate “limbic” brain*, characteristic of all mammals; and (3) *the “neocortical” brain*, characteristic of our species. Recently, several neuroevolutionary theoretical frameworks have developed in line with this view, such as the evolutionary framework of emotions by Panksepp and Solms (2012), the polyvagal theory by Porges (2007), and the neurocognitive model of levels of consciousness by Morin (2006). The elements of the CER model will be briefly outlined, grouped into the following phases: stimulus situation and primary cognition, experiencing and expressing emotion, to highlight the corresponding components with those theories. Awareness as a tertiary cognition will be explored in more detail due to its relevance for emotional literacy.

#### 3.1.1 Stimulus situation and primary cognition

Milivojevic (2000) used the term *stimulus situation* to stress that people respond not to raw environmental inputs but to their interpreted meaning. This process—*primary cognition*—involves three rapid, often unconscious steps: *perception* (forming mental representations from external or internal stimuli), *apperception* (attributing meaning based on one’s frame of reference), and *valorization* (assessing importance according to personal values). *Only stimuli deemed significant trigger emotional responses*, whose intensity reflects the perceived importance. This aligns with Panksepp and Solms’ (2012) concept of primary processes, where subcortical systems generate core emotions, homeostatic drives/motivational experiences, and sensory affects. In moments of change, primary cognition selects adaptive reactions that prepare the body for rapid, sometimes survival-critical responses.

#### 3.1.2 Experiencing emotion

When primary cognition detects a significant change in circumstances, it initiates biological programs—through the autonomic nervous system (ANS) and endocrine system—that prepare the body both viscerally and motorically. The first appraisal is whether the situation affirms or threatens one’s values, prompting *pleasant or unpleasant emotions* (see Figure 2B), which emphasize adaptive function and align with Panksepp’s (2004) primary emotions: pleasant (SEEKING, CARE, JOY, LUST) and unpleasant (FEAR, RAGE, PANIC/GRIEF). These emotional states correspond to specific ANS activations consistent with Polyvagal theory (Porges, 2007), which distinguishes *three evolutionary stages*: *social communication*, mediated by the myelinated ventral vagus activating the parasympathetic nervous system (PNS) in safety; *mobilization*, via the sympathetic nervous system (SNS) for fight-flight responses; and *immobilization*, via the unmyelinated dorsal vagus activating the PNS in extreme threat, producing ‘freeze’ and shutdown. Following James (1884) and Schachter and Singer (1962), Milivojevic (2000) holded that these bodily reactions function to bring emotions into awareness to motivate adaptive action, though individuals may remain unaware of suboptimal responses—a topic explored later in the CER model.

#### 3.1.3 Expressing emotion

Expressing emotion involves a sequence from *motivational preparation to action* (see Figure 2A). Emotional body reactions heighten the drive for specific behaviors, felt as impulses and reflected in posture or facial expression, often awaiting further preparation but sometimes triggering immediate action. This *motivational state reorganizes mental priorities* as *secondary cognition*—engaging thought, perception, apperception, and memory to select adaptive responses—*analogous with secondary processes* as proposed by Panksepp and Solms (2012), linked to associative learning and the limbic system. *Action then aims to restore balance* (see Figure 2B): in unpleasant situations, the goal is to change the situation through active influence (allo-adaptation) or by leaving situations and choosing a more comfortable ones (de-adaptation). If successful, a sense of relief will follow. In unchangeable problematic situations (for instance, when a significant other dies), re-adaptation assumes changes on a mental level within the frame of reference and value system to accept the new reality (auto-adaptation). However, the frame of reference is a relatively rigid structure; it changes slowly and requires time, so those feelings manifest as moods. A typical feeling is sadness, but it could also be disappointment or despair. Pleasant emotions prompt behaviors that tend to stabilize or intensify affirmative situations, moving toward or seeking to repeat pleasant stimuli. This implies improving quality of life (pro-adaptation), which includes elements of allo-adaptation (organizing the environment according to personal wishes) and auto-adaptation (assimilating positive changes into a frame of reference).

### 3.2 Emotional awareness as a tertiary cognition and emotion regulation

Milivojevic (2000) postulated that emotional reactions can occur outside of our awareness—that we may be unaware of perception (via subliminal stimuli and subception), apperception, valorization, emotional bodily reactions, and action tendencies, and that our behavior reflects these emotional processes. This aligns with the challenges noted by Wiens (2006) and Wiens and Öhman (2007), who emphasized the methodological difficulties in accurately assessing stimulus awareness and distinguishing conscious from unconscious emotional processing. Similarly, LeDoux (2014), in *Coming to Terms with Fear*, argued that defensive responses triggered by subcortical survival circuits should not be equated with the conscious feeling of fear, thereby underscoring the distinction between unconscious emotional reactivity and conscious emotional experience. In line with this, the concept of *emotionally-subliminal stimuli*–stimuli that are perceptually visible but whose emotional relevance is not consciously recognized—has been proposed by Frumento et al. (2022) as a useful framework to better understand unconscious affective processing. These stimuli bypass emotional awareness while still influencing physiological and behavioral responses, thus supporting the notion that unconscious emotional processing plays a critical role in everyday behavior and psychopathology—an idea central to the concept of emotional literacy.

This view corresponds with the delineation of the levels of consciousness and self-awareness by Morin (2006), who integrated nine relevant neurocognitive models and proposed four hierarchical levels: (1) *unconsciousness*—being non-responsive to environment or self (like in coma or deep sleep); (2) *consciousness*—focusing attention on the environment and processing incoming external stimuli *without* being aware of mental events that are taking place; (3) *self-awareness*—focussing attention on self, processing internal stimuli, and becoming a reflective observer; and (4) *meta-self-awareness*—being aware that one is self-aware. Therefore, the emotional reactions outside our awareness correspond to the second level of consciousness as Morin (2006) defined and further illustrated: *“When awake and ‘conscious’, one will process information in the environment and respond to stimuli…The organism will be immersed in experience—an unreflective actor in one’s environment. In this perspective, most—if not all—animals possess ‘consciousness’.”* (p.359).

However, emotions at that level of consciousness can be adaptive only in very simple and urgent situations. Most complex social situations demand a synergy between awareness [or self-awareness, as defined by Morin (2006)] and emotional reactions in order to choose the most adaptive action. In this sense, the CER model describes awareness as a tertiary cognition—*as if* a ‘higher level’ of a system gathers information about processes at a ‘lower level’ of the system and attributes the meaning of emotion to the perceived bodily reactions. Only then can a person identify an emotion, express it verbally, and evaluate it critically. Consequently, awareness of an emotional bodily reaction is a precondition for any kind of emotion regulation (inhibition, intensification, or modification) and for the choice of an adequate reaction (Milivojevic, 2000). Furthermore, as Steiner (2003) has demonstrated, we can be aware of our emotions to varying degrees.

Meta-self-awareness, as the highest level of consciousness (Morin, 2006), corresponds with meta-emotions (e.g., feeling ashamed for being afraid) and can indicate higher levels of emotional literacy and regulation, though this is not always the case, as will be discussed further. In the CER model, awareness as tertiary cognition aligns with the *tertiary processes* described by Panksepp and Solms (2012) as higher cognitive functions in cortical regions. These processes receive “bottom-up” emotional influences (relevant information from primary and secondary processes) to perform tertiary cognitive operations that allow evaluation based on past experiences, as well as exert “top-down” regulatory control over cognition and behavior, i.e., emotion regulation.

### 3.3 The continuum of the structural inadequacy of emotional reactions

**Figure 3:**
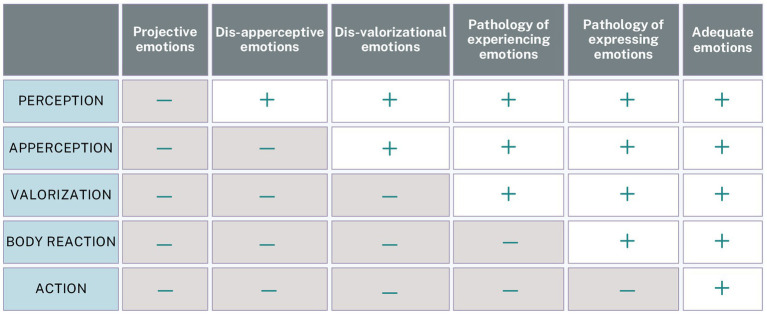
Continuum of structural inadequacy of emotional reactions. The symbol (–) indicates an error in the sequence, while the symbol (+) signifies correctness. Projective emotions: emotional reactions triggered by distorted or fabricated perceptions of reality, including hallucinations (*hallucinatory emotions*), incorrect perceptions of real stimuli (*illusory emotions*), or imagined threats (*phantasmatic emotions*). Dis-apperceptive emotions: emotional responses based on misinterpreted but accurately perceived situations, often due to false beliefs (*delusions*) or lack of information (*cognitive distortions*). Disvalorizational emotions: inadequate emotional responses arising from overvaluing (*hypervalorization*) or undervaluing (*hypo-valorization*) certain personal or social values. Pathology of experiencing emotions: emotional dysfunction characterized by a *lack of, or partial, emotional awareness* (e.g., being aware of only certain aspects or types of emotions) and their misinterpretations (such as somatization, psychologizing, pathologizing, or inadequate metaemotions—e.g., feeling ashamed of being afraid), resulting in *excessive intensity, prolonged duration* (moods), or *suppression* of emotions, and are frequently associated with psychosomatic symptoms and chronic emotional dysregulation. Pathology of expressing emotions: *socially inappropriate* or *ineffective emotional behaviors* that fail to support adaptive responses. Adapted from Milivojevic (2000).

In the CER model, adequate emotions result from emotional reactions satisfactorily progressing through every step. However, ‘mistakes’ can be made at each phase, transferring to the next, which is explicated on the continuum of the structural inadequacy of emotional reactions presented in Figure 3. The closer the ‘initial mistake’ is to the starting point of the circular reaction, the more serious the level of the inadequacy of the emotional reaction, as shown in Figure 3.

Everyone can experience inadequate emotional reactions, with initial mistakes occurring at different points in the sequence; this alone does not imply pathology. However, many people consistently express certain emotions too intensely (excessive emotions) while rarely or never showing others (deficient emotions). These patterns form individual *emotional tendencies or dispositions* (Milivojevic, 2000). In therapy, the goal is to uncover the client’s *specific emotional profile*, as emotional excesses or deficits are often linked to personal challenges or disorders. *Emotion analysis identifies where the “initial mistake” occurs within the CER sequence and examines it across three dimensions: the communication (transactional) field, personality structure, and personal history*. Therapeutic interventions then focus on correcting this specific “error.” Milivojevic (2000) further provided in-depth descriptions of 40 emotions, detailing their structure, functions, communicative roles, healthy and unhealthy expressions, and related therapeutic strategies. Emotion analysis thus serves as the foundation for a transdiagnostic approach to understanding the causes of mental health disorders and guiding their treatment, primarily through the development of emotional literacy and regulation skills.

Trained in transactional analysis by Zoran Milivojevic—and in emotional literacy by Claude Steiner in person as part of that training—the author has rooted her extensive clinical work with over a thousand clients in multicultural contexts in the described emotion analysis, witnessing its profound explanatory and healing power. Additional training in peace studies and systemic family therapy deepened her understanding of the individual’s connection to society and the ecological system. Teaching developmental psychology, differential psychopathology, and qualitative research in an international psychotherapy program further broadened her perspective, and it highlighted key gaps in emotion science—gaps that inspired the research and dissemination of the CER model. However, translating the concept of a “mistake in apperception” into a viable research design proved both elusive and intellectually stimulating. This challenge led to the development of the expanded model of human experience, which is presented in the next section.

## 4 The bio-psycho-social-spiritual-ecological (5D) model of human experience

**Figure 4:**
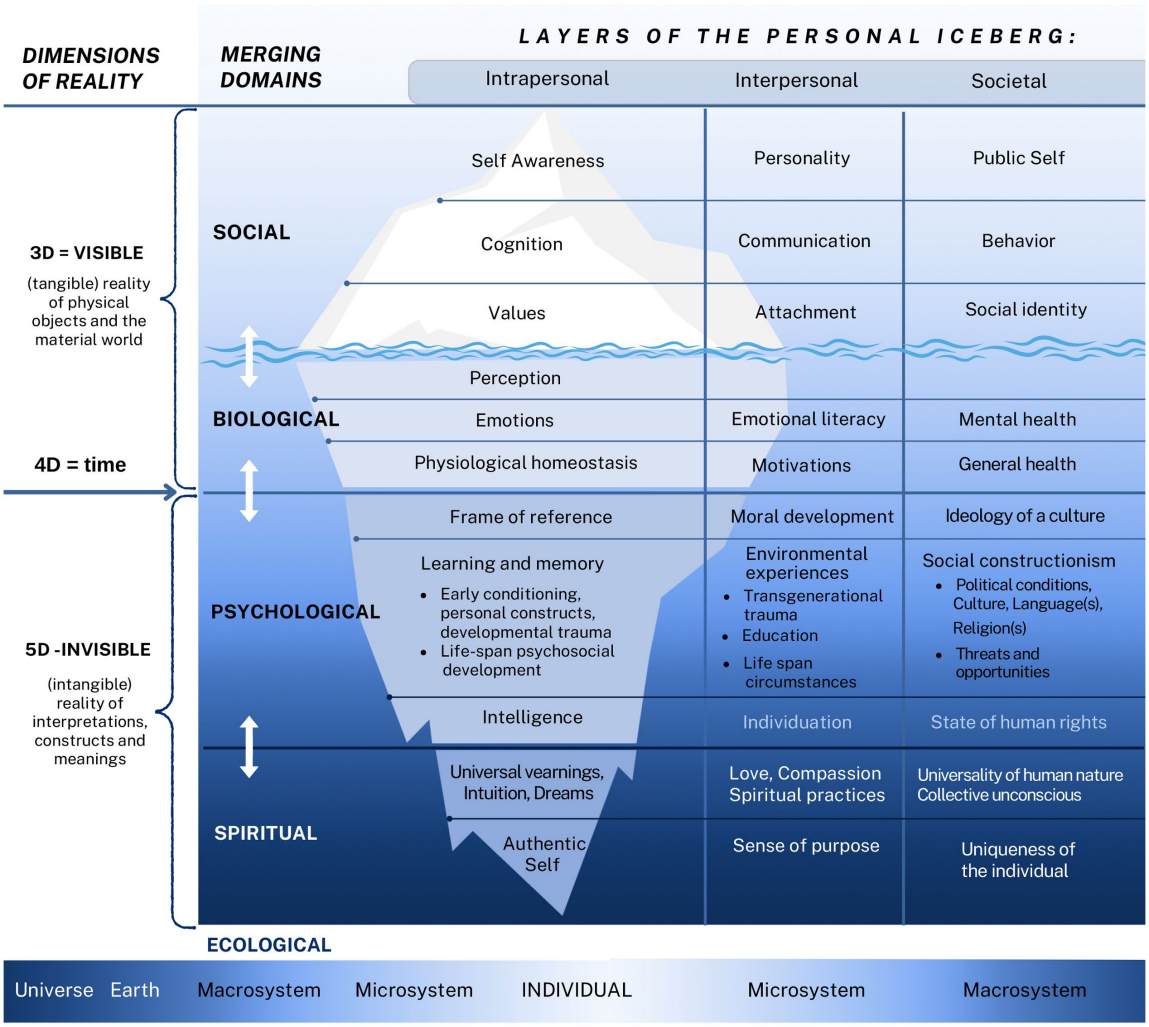
The Personal Iceberg in the Bio-Psycho-Social-Spiritual-Ecological (5D) Model. The Social and Biological domains, as tangible aspects of reality (3D), merge with the Psychological and Spiritual domains, representing intangible reality (5D), across the time dimension (4D). All of this unfolds within the broader Ecological system, influencing personal development at the intrapersonal, interpersonal, and societal levels.

The integration of emotional literacy with the CER model, along with their operationalization, resulted in the development of a novel bio-psycho-social-spiritual-ecological model of human experience—referred to more simply as the 5D model. This model is visually represented as a Personal Iceberg (see Figure 4), where the biological and social domains emerge from the top as tangible and observable elements in the three-dimensional (3D) physical world. In contrast, the psychological and spiritual domains rise from below, representing the more abstract and intangible five-dimensional (5D) realm of internal constructs and meanings. This developmental structure unfolds over time—representing the fourth dimension (4D)—and exists within the broader context of the Ecological system. The bio-psycho-social approach is well established and widely accepted, requiring no further introduction. The inclusion of the spiritual and ecological domains, however, will be briefly explained.

### 4.1 The spiritual domain

*Spiritual* is the adjective form of the word “spirit”—the Latin word for “breath,” meaning the thing that animates life, and as a domain refers to the realms going beyond mere physical existence, including consciousness (Spiritual—Definition, Meaning and Synonyms, n.d.). The evolution of consciousness has been a long, gradual process unfolding over millions of years. According to findings in evolutionary biology, self-awareness—associated with the development of the primate cortex—may have emerged approximately 5 million years ago (Mashour and Alkire, 2014). It then took millions more years for the first known civilization to arise in Mesopotamia, approximately 4,000–3,500 B.C., where sophisticated systems of astronomy, astrology, astral medicine, and other divinatory practices were developed to understand the connection between humans, the divine, and the cosmos (Rochberg, 2014). Since then, cultures across the world have continually sought to understand human nature and the universe through mythology, spiritual traditions, philosophy, religion, science, and art. Across traditions, various terms have been used to describe the force that animates life, such as God, Source, Spirit, Higher Power, Life Force Energy, Prana (in Indian scriptures), and Chi (in Chinese philosophy). This enduring quest reflects the *universality of human nature*, grounded in a species-wide set of complex psychological adaptations (Tooby and Cosmides, 1990).

*In the 5D model, the Spiritual domain refers to the laws of nature and the universe, and their connection to human nature itself*. Despite tremendous scientific advancements, much of the human mind remains unknown—what Jung (1969b) called the *collective unconscious*, the deepest layer of the psyche, containing universal content that influences us without our conscious awareness. Its symbolic expressions appear across belief systems and cultural practices, reflecting universal patterns of emotional and mental life known as *archetypes* (Jung, 1968). Unlike the personal unconscious, which contains forgotten or repressed personal experiences, the contents of the collective unconscious have never been in individual’s consciousness but owe their existence to heredity and primordial mental structures. Jung (1968) described archetypes as “forms without content,” which become conscious secondarily, filled with meaning through individual experience to form various representations of motifs without losing their basic pattern. Through *dreams* and *intuition*, described by Jung (1971) as “perception via the unconscious,” we access its wisdom and archetypal insights.

*Spirituality as a human experience* is defined comprehensively to reflect the *various ways it can be experienced and expressed on intrapersonal, interpersonal, and societal levels*. As Puchalski et al. (2014, p. 646) stated:

*“Spirituality is a dynamic and intrinsic aspect of humanity through which persons seek ultimate meaning, purpose, and transcendence, and experience relationship to self, family, others, community, society, nature, and the significant or sacred. Spirituality is expressed through beliefs, values, traditions, and practices.”*

### 4.2 The ecological domain

Drawing on Bronfenbrenner’s Ecological Systems Theory (1994), this domain encompasses the multilayered environmental systems that influence human development and behavior. It is conceptualized as a nested structure of interrelated contexts, each exerting varying degrees of influence on the individual. These systems include the *microsystem* (e.g., family, school, peer group), the *mesosystem* (interactions among microsystems), the *exosystem* (indirect environments such as a parent’s workplace), the *macrosystem* (broader cultural, economic, and societal structures), and the *chronosystem* (temporal dimensions such as life transitions and historical change). Bronfenbrenner (1994) emphasized that development is shaped by reciprocal interactions between the individual and these environmental layers, which function dynamically over time.

In the 5D model, the ecological domain is *expanded to the global level*, conceptualizing Earth as an interconnected system within the broader context of the universe. This perspective encompasses the vast environmental systems and interdependencies that support life and influence human development across populations and regions. The global ecological domain provides the context within which the biological, psychological, social, and spiritual dimensions unfold. It underscores that human wellbeing is inseparable from the health of the planet, emphasizing the systemic and reciprocal relationship between humanity and the global environment.

### 4.3 The personal iceberg metaphor

The conceptualization was inspired by Satir et al. (1991), who presented the intrapsychic system as an iceberg—where observable behavior appears above the waterline, coping stances are partially visible, and deeper layers include feelings, perceptions, expectations, yearnings, and the self (Banmen, 2002). In the 5D model, Satir’s concepts of *yearnings* and *the self* are retained as the two deepest intrapersonal layers, while the remaining layers have been newly developed. Additionally, each layer is elaborated across the intrapersonal, interpersonal, and societal levels, aligning with Bronfenbrenner’s ecological system: the individual, microsystem, and macrosystem, respectively (see Figure 4).

Given the model’s comprehensive scope, each layer connects to concepts and disciplinary perspectives that extend beyond the focus of a single article. To provide context, concise definitions and summaries of key theoretical foundations—covering the layers above the waterline, and spiritual practices—are presented in Supplementary material in a top-down format. In contrast, the present section adopts a bottom-up, developmental perspective (4D), tracing the interaction of layers over time, particularly in the middle of the iceberg where the 5D (intangible) and 3D (tangible) domains intersect. It begins with the 5D domain, examining *how nature and nurture interact to shape the frame of reference conceptualized as levels of consciousness*, and introduces the *central hypothesis* that these levels are manifested in the 3D realm and directly proportional to emotional literacy, including two proposed advanced stages. This hypothesis is then *operationalized* by proposing that the levels of consciousness/emotional literacy are embodied in the degree of physiological homeostasis, levels of motivational needs and general health, reflected on the continuum of structural inadequacy of emotional reactions and mental health outcome, and expressed on the continuum of observable psychological functions.

### 4.4 The interaction of nature and nurture influences in the 5D realm

The bottom three intrapersonal layers of the 5D model most clearly reflect the influence of nature. Imagine babies born simultaneously around the world—each genetically and biochemically unique (Tooby and Cosmides, 1990), yet all equally worthy, authentic human beings, innately seeking love, acceptance, and validation, with distinct traits and latent talents awaiting development. However, the worlds they are born into differ dramatically.

#### 4.4.1 Environmental circumstances

These infants enter vastly different environments across countries, languages, cultures, ideologies, religions, political systems, economies, climates, and varying levels of safety, resources, or instability (e.g., war, upheaval, or natural hazards). They also encounter differing degrees of respect for human rights and social justice, shaped by the prevailing power dynamics within their societies. *Power relations* take multiple forms—visible, hidden, and invisible—that influence who participates in decision-making and whose voices are silenced (VeneKlasen and Miller, 2007).

Foucault (1977) emphasized that power is embedded in knowledge systems and social norms, shaping what is considered “truth” and legitimizing control over marginalized groups. A relevant example is the phrase “boys do not cry,” which illustrates how social norms and power relations shape identity, behavior, and emotional literacy. This phrase imposes a gendered expectation of emotional repression, defining masculinity as stoic and detached while labeling emotional expression as feminine or weak. According to Foucault (1977), it reinforces a cultural ideal that regulates emotional expression through internalized discipline and social norms. Gergen’s (1991) theory of social constructionism complements this view by asserting that such phrases are not natural or universal truths, but socially constructed through language and reinforced through relationships and discourse.

Within these broader societal contexts, each child is born into a particular family setting, shaped by the caregiver’s own developmental level and capacity to offer secure, nurturing relationships. According to *attachment theory* (Bowlby, 1969), early relationships with primary caregivers form internal working models that guide the child’s expectations of self and others. However, caregivers bring their own histories—often shaped by unresolved trauma, lack of emotional literacy, and life stressors—that influence their ability to respond sensitively to a child’s needs. This process of *transgenerational transmission of trauma* can disrupt the development of secure attachment (Fonagy et al., 1991; Kostova and Matanova, 2024), especially when caregivers lack the internal or external resources to provide consistent, attuned caregiving.

Taken together, these early experiences shape the child’s meaning-making processes, as they construct reality through interactions with their environment. In early life, before cognitive capacities fully develop, *attachment styles* (Ainsworth et al., 1978; Main and Solomon, 1986) and *social constructions* act as a kind of “family and cultural programming”—what Berne (1975) called a *Life Script*, later expanded by Steiner (1994). The unfolding of this process from a neuroevolutionary perspective is briefly outlined in the following section.

#### 4.4.2 The influence of nature

At the very bottom of the iceberg is the *authentic self*, as the unique human being, also known as the *soul*, *being*, or simply the *self* in Jungian terms (Jung, 2006). Recent neuroscientific research affirms that *affects are the primary organizers of subjective experience*. Newborns enter the world with subcortical brain areas already functionally mature, supporting three core components of affect: primary emotions, homeostatic drives/motivational experiences, and sensory affects (Panksepp and Solms, 2012). They refer to the inherited capacity to experience “primary-process sensitivity devoid of any specific content or clear cognitive distinction between the external-objective and the internal-subjective world” (Alcaro et al., 2017, p. 8). According to Alcaro et al. (2017), this *“affective core-self”* integrates perceptual stimuli into a unified, conscious, and intentional state, aligning with Jung’s conception of the self as rooted in subcortical structures that mediate both psychic and physiological processes. Jung emphasized the ontological significance of consciousness, writing: “*consciousness is a precondition of being*.” Thus, the psyche is endowed with the dignity of a cosmic principle, which philosophically and in fact gives it a position coequal with the principle of physical being. The carrier of this consciousness is the individual, who does not produce the psyche on his own volition but is, on the contrary, preformed by it and nourished by the gradual awakening of consciousness during childhood” (1957/2006, p. 37).

*Universal yearnings* refer to the deep, intrinsic human longings—to love oneself and others, to be loved, accepted, and validated (Satir et al., 1991). Underlying those yearnings and human nature is the “orderly universe” and the dynamic spiritual base for that order, which Satir calls the “Life Force.” These yearnings align closely with what Panksepp (2004) identified as the primary emotional systems rooted in the subcortical brain, particularly the SEEKING, LUST, CARE, and PLAY systems. The CARE and LUST systems underpin nurturing behavior and the desire for closeness and bonding; the SEEKING system motivates exploration and connection; and the PLAY system supports joyful social engagement. In this sense, the neuroevolutionary mechanisms of affect provide the biological grounding for what Satir described in psychological and spiritual terms: a universal, life-affirming drive for connection, growth, and love.

*Intelligence* refers to latent mental abilities underlying cognitive behavior. Demetriou and Spanoudis (2017) defined it as “a phenotype interfacing the developing individual with the world,” enabling meaning-making and problem-solving, especially under uncertain conditions. Primarily related to fluid intelligence, it grows with age and experience and, in later stages, supports metacognition and critical thinking—allowing individuals to deconstruct prior knowledge and assumptions and serving as a key driver in the process of individuation.

#### 4.4.3 The intersection of nature and nurture through learning and memory

The subcortical regions involved in the emergence of primary processes powerfully modulate developing secondary processes in the limbic brain via *associative learning mechanisms*—classical (Pavlovian) and instrumental (operant) conditioning (Panksepp and Solms, 2012). The synergy activated in emotional situations between the hippocampus (that plays a crucial role in learning and declarative memories) and the amygdala (that recognizes pleasure, rewards, or threats and prepares the body for fight or flight response) is particularly relevant for memory encoding and the formation of long-term memory. This synergy suggests that emotional content is remembered better than neutral content (Tyng et al., 2017). *Mirror neurons*, which activate during both action and observation (Rizzolatti and Craighero, 2004), provide a neural basis for Bandura (1977) *social learning theory*, illustrating how imitation and modeling shape behavior and emotional understanding—processes that, over time, inform and reinforce tertiary cognitive functions like thinking, planning, and reasoning. These higher processes develop through learning and memory and are structured by early-formed *personal constructs* (Kelly, 1991) and *internal working models* (Bowlby, 1969), which, especially when formed in preverbal stages, can become deeply embedded and resistant to change, often perceived as objective truths rather than constructed interpretations.

*Taken together, affective states, associative learning, personal constructs and social learning can be regarded as a circuit of personal experience*. Alcaro et al. (2017) proposed that all accumulated personal experiences “during individual history take the form of clusters (or complexes) of perceptual memory traces gravitating around an affect” (p. 9), aligning with Jung’s concept of *“feeling-toned complexes.”* Jung (1969a) described complexes as psychological structures composed of various mental contents and representations united by a common emotion (e.g., the inferiority complex comprises thoughts, memories, and fantasies related to a lack of self-worth). These complexes further influence how new emotional situations are interpreted and experienced during development, often intensifying in adolescence. From the onset of puberty, the brain “rewires” itself—particularly in the prefrontal cortex, which reaches full maturation at approximately 25 years of age (Arain et al., 2013)—a development critical for emotion regulation. Ongoing psychosocial development across the lifespan, as described by Erikson (1994), is shaped by early experiences, constructs, and complexes; however, higher cognitive capacities for reflection and emotional regulation become increasingly available over time.

### 4.5 Levels of consciousness as an outcome of nature and nurture intersection

The intersection of nature and nurture in the 5D domain results in the development of a *frame of reference*—a personal set of beliefs about the self, others, and the world (Milivojevic, 2000), serving as an internal “map” for interpreting life experiences. The formation of this belief system depends on cognitive and emotional development, and it is reflected interpersonally through corresponding levels of moral development shaped by the sociocultural environment. Kohlberg, (1984) theory outlined three major levels of moral reasoning—each with two stages—and later added a seventh, describing how individuals relate to social rules and expectations over time. A key intrapersonal dimension of the frame of reference introduced by the 5D model is the *locus of power*—beliefs about where power resides and how it is distributed among self, others, and the world. Power, as VeneKlasen and Miller (2002) noted, is “both dynamic and multidimensional, changing according to context, circumstance, and interest,” and can manifest as domination, resistance, collaboration, or transformation (p. 39). The 5D model outlines four developmental stages of the frame of reference, which evolve alongside moral development from infancy to adulthood. *Each stage represents a distinct level of consciousness, offering a unique lens for interpreting information with varying degrees of complexity and accuracy*. While *progression through these stages reflects a natural developmental path*—beginning with children’s inherent dependence on caregivers and limited personal agency—*this development can become arrested at earlier stages due to suboptimal environmental conditions*, particularly insecure or disorganized attachment. The following section briefly introduces each stage, focusing on how it manifests in adulthood, and linking it to the corresponding moral stage and perceived locus of power. The relationship to attachment patterns will be explored in later sections.

#### 4.5.1 Self-centered level of consciousness—“I vs. Others”

As the name suggests, this stage is characterized by an egocentric perspective—an aspect of the preoperational stage of cognitive development described by Piaget (1971)—reflected in an inability to consider situations from other people’s perspectives. Moral development is at the *preconventional level*, where rules and social expectations are viewed as external to the self. In the first stage, behavior is guided by the desire to avoid punishment, and in the second, by the desire for reward. A person in the concrete operational cognitive stage is typically limited to this level of moral reasoning (Kohlberg, 1984). Power is perceived as personified, expressed through status and wealth, and distributed among people in a “zero-sum game.” There can only be winners and losers, and the resulting relational logic often follows the belief: “If I do not control, I will be controlled.” VeneKlasen and Miller (2002, p. 39) referred to this form of power as “power over.”

#### 4.5.2 The collective level of consciousness—“Us vs. Them”

The collective level corresponds with the *conventional level* of moral development, where the self begins to identify with, or internalize, the rules and expectations of others—particularly those of authority figures within the society (Kohlberg, 1984). In the third stage, behavior is guided by the desire to be seen as a “good boy/girl,” while in the fourth stage, it is governed by adherence to the law and societal order, often without critical examination of those rules and values. As such, individuals functioning at the lower range of the formal operational cognitive stage are typically limited to this level of moral reasoning. As the term conventional suggests (i.e., conformist, conservative, standard), most people reach—and remain at—this stage. Social identity theory (Tajfel and Turner, 1979), particularly its insights into the “Us vs. Them” mentality and in-group favoritism, is especially relevant here. Power is attributed to authorities or leaders, often accompanied by a subjective sense of *learned helplessness* (Seligman, 1972) that can lead to passivity, conformity, or dependency on external structures. Resources are viewed as scarce and distributed within a competitive, zero-sum framework—but now on a collective scale: e.g., “They are taking our jobs, land, and resources.” Importantly, this level involves not just material competition but also identity-based threats: “They are inferior, dangerous, or undermining our values,” or “They are threatening the very fabric of our society.”

#### 4.5.3 Individual level of consciousness—“I and Others”

This level corresponds to the *postconventional level* of morality, where the individual has differentiated self from the rules and expectations of others and defines their values in terms of self-chosen principles (Kohlberg, 1984). Abstract (formal operational) and critical thinking are necessary at this level, leading to the realization that rules and expectations exist as part of a social contract (stage five), but that these rules are relative and subject to change. This stage involves a process of deconstruction—for example, recognizing that culture itself is a construct and that conflicts arise not between cultures, but between individuals (Mehta and Rückert, 2004). Stage six is characterized by values such as social justice, the universality of human rights, equity, and equal respect for all people (Kohlberg, 1984). The sense of power here comes from awareness of and respect for one’s own and others’ rights, grounded in empowerment and emancipation. In line with transformative paradigm and critical theory, cultural relativism is rejected, acknowledging that various privileged versions of reality and knowledge are socially and historically situated (Mertens, 2007) leading to various forms of abuse of power, injustice, and oppression, “which demands an engagement with the suffering of the people of the lived world” (Steinberg and Kincheloe, 2010, p. 149).

#### 4.5.4 Unity consciousness—“Us”

The sixth stage of the postconventional level is already underrepresented in the population and therefore remains insufficiently researched (Kohlberg, 1984). However, Kohlberg proposed a seventh stage, which is often omitted in textbooks. This seventh stage is characterized by the development of ethical and spiritual thinking, which “culminates in a synthetic, nondualistic sense of participation in, and identity with, a cosmic order…From such a framework, moral principles are not seen as arbitrary human inventions; rather, they are seen as principles of justice that are in harmony with broader laws regulating the evolution of human nature and the cosmic order” (Kohlberg, 1984, p. 250). Perhaps this view is most easily grasped when envisioning Earth from the orbital perspective (Garan, 2015), where it becomes clear that “Us” refers to the human species—a spectacular manifestation of life and intelligence that has emerged through billions of years of evolution. From this vantage point, it becomes evident that we should cherish and protect our planet, with its extraordinarily rare and beautiful conditions for life. Yet, by focusing on arbitrarily constructed human differences and striving for control, we perpetuate suffering and fuel endless wars across the globe. At this stage, power is understood as a natural or cosmic force—something we are part of, and therefore something that resides within us. This concept will be further elaborated in the next section.

### 4.6 Central hypothesis: levels of consciousness are directly proportional to emotional literacy

**Figure 5:**
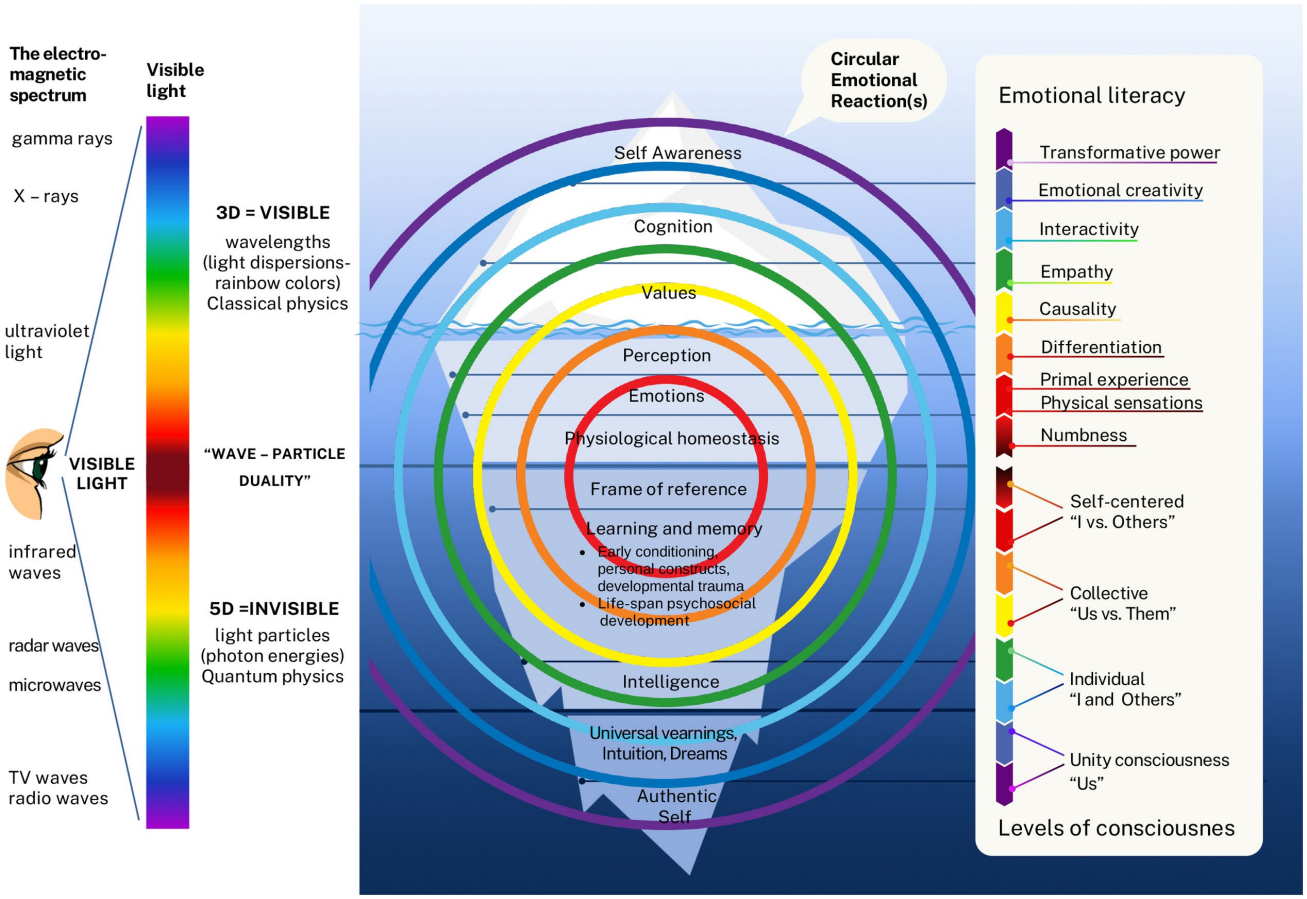
Emotional Literacy and Levels of Consciousness in the 5D Model. Drawing an analogy with the “wave–particle” duality in physics—where visible light exhibits both wave and particle properties at the same frequency (as seen in the colors of the rainbow)—the 5D model proposes that emotional literacy is directly proportional to levels of consciousness, represented here by the same color spectrum. The model further suggests that a self-centeredlevel of consciousness corresponds with numbness, physical sensations, and primal experiences; the collective level with differentiation and causality; the individual level with empathy and interactivity; and unity consciousness with emotional creativity and transformative power.

In the previous section, it was shown that levels of consciousness develop over time (4D), shaped by the dynamic interplay of nature and nurture within the intangible 5D realm—where affective and cognitive processes evolve together. Under suboptimal environmental conditions, this development can be arrested at lower levels of consciousness. *The core hypothesis of the 5D model posits that the proposed levels of consciousness from the 5D realm are directly proportional to emotional literacy as expressed in the 3D physical reality.* This relationship is illustrated in Figure 5.

This hypothesis conceptually aligns with Einstein’s famous equation, E = mc^2^, which reveals that energy and mass are two forms of the same essence, resonating at a shared frequency. Within the 5D framework, the 5D realm represents the energy field—encompassing information processing and electromagnetic activity—while the 3D reality reflects its physical manifestation. The model draws on wave-particle duality^1^ to further explain this relationship: just as light and energy can exhibit both particle- and wave-like properties, emotional and cognitive processes can simultaneously manifest in energetic and material dimensions. Central to this model is the CER—as previously discussed, an adaptive mechanism involving visceral, motoric, motivational, and mental processes. The 5D model links levels of consciousness with emotional literacy through CER, symbolized in distinctive rainbow-like colors that reflect both frequency and the functional unity of body and mind. This feedback loop reflects how all physiological and psychological functions operate in unison, oriented toward growth and adaptation. Emotions, in this view, can be conceptualized as *E-motion*—energy in motion, resonating on different levels of frequency.

Errors can occur at any phase of the CER cycle, influencing subsequent stages. The closer an error is to the initial point in the circular reaction, the deeper the potential psychological disturbance, manifesting in alignment with levels of consciousness and emotional literacy, each associated with a specific frequency or “color” in the model. *The 5D model further proposes that a self-centered level of consciousness corresponds with numbness, physical sensations, and primal experiences; the collective level with differentiation and causality; and the individual level with empathy and interactivity*. Responding to Steiner’s (2003) invitation to conceptualize higher levels of emotional literacy, the 5D model introduces *emotional creativity* (Averill, 1999) as a hallmark of the *unity level of consciousness*. This construct reflects the capacity to access the deepest layers of the personal “iceberg”—the spiritual domain—leading to the discovery of the authentic self and a sense of purpose. Ultimately, *transformative power* is proposed as the highest expression of emotional literacy: the ability to live purposefully and in alignment with the self, the world, and all the colors of the rainbow.

#### 4.6.1 Emotional creativity (EC)

EC construct, operationalized through the Emotional Creativity Inventory (ECI) (Averill, 1999), comprises three components: understanding and learning from one’s own and others’ emotions, the ability to experience unusual emotions, and the capacity to express emotions effectively and honestly. A meta-review of studies using the ECI (Kuška et al., 2020) showed positive correlations with personality traits such as warmth, positive emotions, openness to experience, and neuroticism; creative dispositions; the escape-avoidance coping strategy; and negative correlations with alexithymia. Recent developments in the construct (Trnka, 2023) suggest that these personal dispositions serve as underlying “prerequisites” of EC, leading to outcomes such as greater awareness of the potential benefits of emotions, improved social interactions, a more authentic sense of self, enhanced emotion regulation, and increased creative performance.

#### 4.6.2 Transformative power

The highest expression of emotional literacy and unity consciousness is best described through the definition and example of VeneKlasen and Miller (2007). They have developed an action guide for advocacy and citizen participation that combines a sound theoretical foundation and concrete practical steps—and implemented them to help establish a network of organizations for justice and empowerment, especially in Africa and South America. They have defined transformative power as: “power relationships that reject the domination and exclusion of some persons by others. Transformative power grows from respect for self and equality with others—their diversity of identity, experience, and ability. It is the power inside us (“power within”), the collective power together with others (“power with”), our ability to speak out and act (“power to”), and the power to work for the change we want (“power for”). These alternatives offer positive ways of expressing power that enable us to create more equitable relationships and structures and to transforming power over” (Sources of Transforming Power, n.d., p. 3).

The central hypothesis is further *operationalized by proposing that levels of emotional literacy*—used interchangeably with levels of consciousness—*are embodied in the degree of physiological homeostasis*, levels of motivational needs, and general health; *reflected along a continuum from structural inadequacy of emotional reaction*s and mental health outcomes; and *expressed along a continuum of observable psychological functions*. This will be elaborated in the following sections.

### 4.7 Emotional literacy as embodied degree of physiological homeostasis, motivational needs, and general health

As outlined in the CER model, *emotion functions as a feedback system aiming to restore equilibrium*. Primary cognition constantly monitors the external (perception) and internal (interoception) environment, relative to a person’s frame of reference. When significant changes are detected, it triggers emotional body reactions—visceral and motor responses intended to adapt to the situation. These reactions align with Polyvagal Theory (Porges, 2007), which can be illustrated by the metaphor of driving: the ANS operates like a car’s gas pedal (SNS for mobilization), footbrake (ventral vagus PNS for calming), and handbrake (dorsal vagus PNS for immobilization). When threatening situations aren’t resolved or emotional energy is not discharged, that energy may remain stuck in the body, manifesting as trauma, like driving with the handbrake on. Conversely, when threats are successfully navigated and emotional responses complete their cycle, homeostasis is not just restored but potentially enhanced—akin to a smoother, more joyful ride.

On an interpersonal level, *the degree of homeostasis achieved reflects one’s current motivational needs*. Maslow (1943) proposed a hierarchy of needs: physiological, safety, love/belonging, esteem, and self-actualization. While these needs can coexist, self-actualization becomes fully accessible only after foundational needs are met. In later writings, Maslow (1971) introduced *self-transcendence*—a level beyond self-actualization, involving *peak experiences* and commitment to greater causes. Yet, like Kohlberg’s rarely acknowledged seventh stage of moral development, this highest tier is often omitted from mainstream psychology, despite its profound theoretical significance (Koltko-Rivera, 2006).

Aligned with the central hypothesis, each level of consciousness reflects a corresponding stage of physiological homeostasis. These will be briefly explored in terms of attachment styles (Ainsworth et al., 1978), developmental trauma (van der Kolk, 2005, 2015), personality organization as delineated by Kernberg (2004), motivational needs, and dominant emotional patterns—using the car-driving analogy to illustrate each level.

#### 4.7.1 Self-centred level of consciousness—focus on physiological needs and safety

Children exposed to severe developmental trauma—especially when caregivers are the source of fear—respond with PANIC/GRIEF that triggers survival “freeze” reactions. To maintain attachment to those they depend on, they must dissociate from these overwhelming emotions, leading to a chronic defensive state rooted in fear. Over time, they continue to “drive with the handbrake on,” developing disorganized attachment and moving through life unaware that something is fundamentally off, never having experienced what it means to feel safe and loved for who they are. If both emotional and cognitive development are inhibited, this *may result in psychotic or low borderline personality organization*. In contrast, when cognitive skills are encouraged, but emotions are dismissed or ridiculed, narcissistic personality traits often emerge. These individuals, cut off from their authentic self and stuck in a defensive mode rooted in primal fear, seek validation through status, control, and material success—though no amount of wealth and power can fill the inner void. When the LUST system activates in puberty (Panksepp, 2004), the initial thrill of pleasure may offer distraction, but novelty fades. Over time, this can lead to increasingly promiscuous, deviant, or even perverse behavior in an attempt to recapture the original excitement.

#### 4.7.2 Collective level of consciousness—focus on belonging/love

Children with *insecure attachment*—whether through consistent neglect (avoidant) or inconsistent care (anxious)—learn to “pull the handbrake” in response to separation distress. Though capable of joy and connection, they remain subtly aware that something holds them back emotionally, yet they do not know how to release it. Deep down lies *unresolved shame*—without the ability to separate self from behavior, they internalize early wounds as proof of unworthiness. Believing their emotions and self-worth depend on something external, they adopt a black-and-white worldview, characteristic of *high-functioning borderline personality organization*—*or neurotic* structure when cognitive development and defenses are more mature. Their unmet emotional needs lead to inner turmoil, impulsivity, and various *addictive coping strategies*. In their longing for rescue and belonging, they become susceptible to manipulation—especially by self-serving individuals or groups—falling into “divide and rule” dynamics that redirect blame outward, while those in power quietly benefit.

#### 4.7.3 Individual level of consciousness—focus on esteem and self-actualization

Secure attachment enables individuals to explore, develop skills, and gain confidence—learning to “drive,” “read the instrument panel,” “adjust the pedals,” and follow the “traffic rules” of life. Witnessing others’ struggles, many take on the role of the “*mechanic*,” deriving esteem and value from *being needed*, reflecting a neurotic organization. Over time, some realize that taking over the responsibility of others is not only “*burning their fuel”* beyond their limits but also is *reinforcing dependency and preserving status quo*. Turning inward, they begin to choose their own paths, cultivate their potential, and form mutual, meaningful connections. They *navigate life with greater self-direction* and contribute in ways that honor both self and others—recognizing that while social rules may be culturally relative, fundamental human rights are universal. This shift reflects healthier functioning and peaceful coexistence.

#### 4.7.4 Unity consciousness—focus on self-transcendence

At this stage, individuals embark on the process of *individuation*, described by Jung (1968) as the integration of the conscious and unconscious self toward inner wholeness. This journey mirrors the *Hero’s Journey* (Campbell, 1949), where life’s challenges become the “call to adventure,” prompting *reflection not just on direction, but on purpose*. As they cross into unfamiliar inner territory, they confront the *shadow*—the denied or repressed parts of the self—and begin to deconstruct limiting beliefs and false identities. In doing so, they also awaken and integrate the *Anima* or *Animus*—restoring balance between inner intuition and outer expression, feeling and reason, being and doing. Through this initiation, *love is rediscovered* not as something to chase, but as a unifying inner force, a source of meaning and authenticity—*a fuel for our journey*. Hardships become gateways to resilience and deeper awareness. While early attachment is essential for safety, bonding, and survival, it should evolve as psychological maturity grows. *Nonattachment* represents this developmental progression—not emotional detachment, but a state of equanimity and freedom from clinging or fear of loss. Individuals *begin to relate from a place of inner wholeness* rather than lack, cultivating relationships grounded in presence rather than possession, love rather than need, and interdependence rather than control (Sahdra et al., 2010). In the final stage—*the return*—they bring back the insight gained through inner transformation, *sharing it with the world in a spirit of renewed purpose*. Guided by an inner “*navigation system*,” they move toward destinations that feel truly compatible, traveling alongside those who journey well with them, and *savoring the ride*. In this way, *love becomes a state of being*, and life unfolds as a shared *path toward truth, unity, and authenticity*.

#### 4.7.5 Measuring physiological homeostasis

Physiological homeostasis can be measured in many ways, with heart rate variability (HRV) being a key marker of ANS adaptability and both baseline and moment-to-moment physiological and emotional states (Shaffer and Ginsberg, 2017). By capturing the interplay between the sympathetic and parasympathetic branches, HRV provides insight into regulation under stress, rest, and during emotional arousal. Trauma-informed approaches—including Polyvagal Theory (Porges, 2022), Somatic Experiencing (Levine and Mate, 2010), and developmental trauma frameworks (van der Kolk, 2015)—highlight HRV as a marker of resilience and nervous system flexibility. As McCraty et al. (2009, p. 20) noted, HRV reflects heart–brain interactions and ANS dynamics mediated by neural pathways between the two systems. A meta-review of 140 studies (Alvares et al., 2016) found consistently reduced HRV in psychiatric disorders—most notably in psychotic disorders, followed by substance dependence, mood disorders, and anxiety—suggesting a transdiagnostic physiological signature of dysregulation and an early indicator of psychological distress.

#### 4.7.6 General health

Inadequate emotion regulation is closely tied to dysregulated physiological processes, contributing to a wide range of health disorders—likely beyond what is typically classified as psychosomatic. Research by McCraty et al. (2009) suggested that the *heart’s electromagnetic field functions as a systemic integrator*, acting as a modulated carrier wave that communicates information throughout the body and even between individuals. This view aligns with emerging *biofield science*, which proposes that a subtle, organizing energy field—often electromagnetic in nature—regulates biological processes from the cellular to the organismal level (Rubik et al., 2015). Together, these perspectives point to the need for integrative models linking emotional, mental, and physiological health through shared regulatory systems.

### 4.8 Emotional literacy as reflected on the continuum of structural inadequacy of emotional reactions and mental health outcome

Just as Einstein’s (1920) *theory of relativity* holds that time is relative to the observer’s frame of reference, *emotions are likewise temporally relative*—memories or imagined futures can evoke genuine emotional experiences in the present, reflecting the mind’s subjective sense of time. In the CER model, emotions arise from changes in stimulus situations, consciously (perception) or unconsciously (subception) *registered as mental representations, always relative to the individual’s frame of reference*—whether drawn from external stimuli (percepts), internal sources (memories, imagination, inner talk), or associative links (via conscious association or unconscious projection). These representations trigger apperception and valorization processes, forming the basis of every emotional reaction. Errors can occur at any stage of this sequence, and the earlier they arise, the greater their impact on psychopathology; when such errors are systematic and persistent, they create characteristic, enduring emotional dispositions (Figure 3). The following section examines these dispositions at each level of consciousness, defined by the stage in the CER sequence where the “initial mistake” occurs on the continuum of structural inadequacy, and outlines their corresponding mental health outcomes.

#### 4.8.1 Self-centered level –projective and dis-apperceptive emotions

At this level, early dissociation and primitive defenses keep emotional literacy underdeveloped—limited to numbness, vague bodily sensations, or chaotic emotions like panic and rage. These reflect projective and dis-apperceptive emotional processes. *Projective emotions* arise when individuals misperceive or fabricate stimuli. In psychosis, hallucinations can trigger full emotional reactions, while in milder cases, real stimuli are distorted or internal worries fuel imagined threats. *Dis-apperceptive emotions* result from misinterpreting actual situations due to false beliefs (delusions), missing context, or rigid perspectives—leading to inappropriate emotional responses. These distorted processes disrupt emotional regulation and often underlie severe psychopathologies, including *psychosis, severe personality disorders, and narcissistic spectrum conditions.*

#### 4.8.2 Collective level—dis-valorizational emotions and pathology of experiencing emotions

At this level, emotional literacy includes the ability to differentiate emotions and recognize causal links with external events. However, values are often misplaced—emotions are overly influenced by social approval and external judgment. Responsibility is projected outward, leading to a victim mindset. Empathy, if present, is typically limited to one’s in-group, while others are dehumanized. *Dis-valorizational emotions* emerge from distorted personal values shaped by social conditioning. Overvaluing the opinions of influential figures—such as a parent, teacher, or authority figure—can provoke excessive emotional responses (e.g., anxiety over minor criticism), while consistently undervaluing one’s own perspective may lead to dangerous indifference, such as ignoring personal boundaries or needs. Pathology of experiencing emotions includes *suppression*, *intensity dysregulation, and prolonged emotional states* (e.g., moods), as well as *partial awareness of emotions* (when someone is aware only of one or a few sequences of the CER model, instead of all, which full awareness implies) and *misinterpretation of their adequacy and meaning*. When chronic, these dysfunctions contribute to psychosomatic conditions and impaired emotion regulation—manifesting as *mild personality disorders, addictions, affective disorders, or psychosomatic symptoms*.

#### 4.8.3 Individual level—pathology of expressing emotions and adequate emotions

At this stage, individuals become more attuned to their emotions and understand their origins, fostering natural empathy and openness to emotional connection. However, difficulties often emerge in the expression of emotions. When emotional reactions result in *impulsive*, *socially inappropriate*, or *ineffective behavior,* expression fails to support healthy adaptation. Social conditioning—particularly within patriarchal norms—can additionally distort this process: women may suppress anger due to its association with aggression and expectations of submissiveness, while men often repress sadness and fear, seen as signs of weakness in traditionally dominant roles. These patterns frequently manifest in *anxiety and other neurotic disorders*. By moving beyond stereotyped gender roles and cultivating assertive communication, conflict-resolution skills, and relationships based on mutual respect and reciprocity, individuals can express emotions more effectively and advance their emotional literacy to higher levels.

#### 4.8.4 Unity consciousness—adequate emotions and intuition

At this level, individuals experience emotions as refined and trustworthy—no longer distorted by reactive patterns or external conditioning. This marks the emergence of *emotional creativity*, the ability to feel deeply, discern nuances, and respond authentically. With this inner coherence, perception expands inward through what Jung called *“perception via the unconscious”*—opening access to *intuition* (often experienced as gut feelings) and to *dreams* and symbolic imagery (linked to the pineal gland as a metaphorical “third eye”). In Jungian terms, EC corresponds to the *Anima*—the feminine, intuitive energy that attunes us to the inner world and allows for discernment. From this foundation, the *Animus*—the masculine, action-oriented force—can guide aligned, intentional action. When these energies are integrated, emotional insight becomes a compass for life, while transformative power enables one to live in harmony with the self and the greater whole. This balance leads to greater *wellbeing*, *clarity of purpose*, and an authentic expression of unity consciousness.

### 4.9 Emotional literacy as expressed on the continuum of observable psychological functions

The described levels of consciousness are reflected in observable psychological functions, depicted in Figure 4 as the iceberg layers above the waterline. Table 1 outlines their continuum at the intrapersonal, interpersonal, and societal levels, corresponding to the levels of consciousness and arranged in a “bottom-up” sequence following the previously introduced layers, presenting together the continuum of the integrative developmental model. These hypotheses draw on 15 years of applying emotion analysis in psychotherapy with over a thousand clients in multicultural settings—effectively representing the outcomes of longitudinal, qualitative, and process-oriented research.

**Table 1:** Continuum of psychological functions and physiological homeostasis on different levels of consciousness in the 5D model.

Psychological functions	Levels of consciousness			
Frame of reference	Self-Centered “I vs. Others”	Collective “Us vs. Them”	Individual “I and Others”	Unity consciousness “Us”
Moral reasoning (Kohlberg)	Preconventional	Conventional	Postconventional	Cosmic order
Motivation (Maslow)	Physiological needs, Safety	Belonging, Esteem	Self-actualization	Self-transcendence
Physiological homeostasis [e.g., heart rate variability (HRV)]	Significantly reduced HRV	Reduced HRV	Average HRV	Increased HRV
Emotional literacy (Steiner)	Numbness, Physical sensations, Primal experience	Differentiation, Causality	Empathy, Interactivity	Emotional creativity, Transformative power
Emotions (CER—Milivojevic)	Projective, Dis-apperceptive emotions	Dis-apperceptive, Dis-valorizational emotions, Pathology of experiencing emotions	Pathology of expressing emotions, Adequate emotions	Adequate emotions
Mental health	Psychosis, Severe personality disorders, Narcissistic spectrum	Mild personality disorders, Addictions, Affective disorders, Psychosomatics	Anxiety disorders, Mental health	Wellbeing
Values (Schwartz—third order values)	Self-enhancement	Conservation	Openness to change	Self-transcendence
Attachment styles (Bowlby/Ainswort)	Disorganized	Avoidant/Anxious	Secure	Nonattachment, Love, Intimacy
Cognition (Piaget/Kuhn/Maslow)	Concrete operational/Formal	Concrete operational/Formal	Formal/Critical thinking	Critical thinking/ Being cognition
Communication styles	Aggressive/ Manipulative	Passive/ Passive-Aggressive	Assertive/ Compromising	Collaborative/ Transformative
Behavior (Bern/Karpman)	Persecutor	Victim	Rescuer/ No games	Authenticity/ Intimacy
Self-awareness	Distorted self-awareness, dissociations, denial, dominant personal unconsciousness	Lower self-awareness, repressed traumatic events, dominant subconscious	Self-aware, reflective, remained biases and “blind spots”	Meta self-awareness, highly reflective
Personality (Freud/Jung)	dominant Id	dominant Super Ego	dominant Ego	Self
Individuation (Jung)	Persona	Shadow	Anima/Animus	Self

#### 4.9.1 Visible layers of the iceberg

The findings are conceptualized in relation to established, time-tested theories with proven transcultural validity. At the values level, Schwartz’s (2012) theory of basic human values is most relevant to the 5D model, identifying 10 values recognized across cultures and grouped into four third-order categories. What we value and whom we are drawn to connect with the previously discussed attachment styles at the intrapersonal level and to social identity at the societal level, as described in Tajfel and Turner’s (1979) social identity theory. At the cognitive level, Piaget (1964) theory of cognitive development is expanded with Kuhn (1999) metacognitive skills and Maslow’s (1971) concept of Being Cognition as the highest form of cognition. On the interpersonal level, cognition manifests through communication (Lazarus, 1973) and conflict management styles, and is observable in behavior—most notably in Berne (1964), further explained by Karpman’s (1968) drama triangle, where the roles of Persecutor, Victim, and Rescuer capture emotional reversals and sudden shifts. At the level of self-awareness, the proposed stages draw on Morin’s (2006) neurocognitive stages of consciousness, further reflected at the personality and public-self levels in relation to the classic theories of Freud and Jung. A concise overview of these theories, along with definitions of the respective stages introduced in Table 1, is provided in Supplementary material.

#### 4.9.2 The continuum of developmental outcomes

The summary of hypotheses in Table 1 is intended to illustrate a “prototypal profile” for each level of consciousness. In reality, development is far more continuous and variable, with some domains advancing more than others. The process of change and growth is similarly non-linear. Typically, the first shift occurs as an insight that alters one’s values, providing motivation for new directions. This is followed by the adoption of new behaviors, which precede changes in emotional dispositions and, ultimately, the establishment of a new level of physiological homeostasis. The model assumes that all humans possess the potential to reach a unity level of consciousness, but its realization depends on supportive conditions; adverse environments and traumatic experiences at any scale or stage of life can hinder this development. The timing of these adversities is critical, with the earliest experiences being the most impactful, as reflected in the importance of attachment styles and their influence on the trajectory of development. Nonetheless, this should not be interpreted deterministically. Human development is marked by significant resilience and plasticity, with opportunities for growth across the lifespan through *healing, individuation, and spiritual practices* (outlined in Supplementary material), and other transformative processes.

#### 4.9.3 Universal developmental patterns across cultures and history

The 5D model identifies universal developmental patterns shared across cultures and history, reflecting a common human nature rooted in species-typical psychological adaptations, despite individual genetic uniqueness and cultural diversity (Tooby and Cosmides, 1990). Cross-cultural studies in 82 countries reveal a broad consensus on 10 basic personal values, ranking those related to self-transcendence highest and those related to self-centeredness lowest, suggesting widely shared aspects of human nature (Schwartz, 2012). Joseph Campbell’s Hero’s Journey, influenced by Jung’s archetypes, showed how myths and religious narratives worldwide share symbolic parallels tracing the human journey from infancy to maturity. While acknowledging cultural differences, Campbell focuses on revealing deep common truths—much like anatomy textbooks highlight shared structure over variation—pointing toward unity through recognition of our shared humanity, echoing the Vedic maxim: “Truth is one, the sages speak of it by many names” (Campbell, 2004, p. XXII). Leonardo da Vinci’s *Vitruvian Man* captured all these messages in an iconic symbol often read as both a literal anatomical study and a metaphor for the unity of body and spirit, the earthly and the divine—a diagram of proportion that became a symbol of human potential. In this reading, the square-centered figure anchored at the genitals reflects the material ground of life, symbolizing a self-centered level of consciousness, while the circle-centered figure at the navel—understood in traditional healing as an energetic center—embodies full development to wholeness and unity consciousness.

## 5 Emotion regulation in the 5D model

In the 5D model, emotion is viewed as energy in motion—resonating at varying frequencies, shaped by feedback loops, and relative in time. Adequate emotion is available energy directed toward action in the right direction, grounded in love for oneself and others. *Emotion regulation is therefore key to redirecting inadequate emotions by adjusting the energy generated, reassessing the accuracy of interpretation, and selecting effective, appropriate actions*. To better understand this process, it is helpful to revisit Figure 2 through the lenses of emotional literacy.

Figure 2A illustrates a rapid cycle in which steps before mental preparation occur within a second, beyond conscious control. However, their purpose is to trigger emotional bodily reactions that bring the emotion into our awareness—an essential prerequisite for emotion regulation. At this point, we can ask the critical question: *Have I interpreted the situation correctly?* This question alone engages tertiary, analytical processes and hands “control” over to our higher functions, which can help calm the reactions immediately. If needed, breathing techniques, physical activity, meditations and other spiritual practices can help calm the activated energy and ground the person in the present time.

From here, the algorithm in Figure 2B supports further inquiry: How is power distributed? Does my perception reflect present reality or echo childhood experiences? What is realistically changeable? What truly affirms me—meeting others’ expectations or aligning with my authentic self? Interpretations vary greatly with levels of consciousness, power dynamics, and concepts of justice, often contrasting sharply between child and adult perspectives and revealing areas for healing. Finally, we ask: What action is appropriate, effective, and fair? How can we protect our rights while respecting others? Addressing these questions fosters the development of key interpersonal skills, such as assertive communication and conflict management.

In this way, *emotion-regulation skills* involve mental reflection to challenge distorted beliefs or frames of reference—a process known in adult education *as transformative learning*, which serves as *a critical mechanism for development toward higher levels of consciousness*. Mezirow (Mezirow, 2000) described transformative learning as a reflective process in which we revise our taken-for-granted frames of reference to become more inclusive, discerning, open, and reflective, a change that emerges through dialogue among diverse learners and fosters both cultural pluralism and social justice. Through various forms of experiential learning, education, and training, individuals can be empowered, transformed, and significantly enhance their emotional literacy—serving both preventive and corrective purposes with the support of mental health professionals. To realize this potential, principles of critical pedagogy, transformative learning, inclusive definitions of culture, and idiographic approaches should be regarded as essential components of mental health education and training (Dragic et al., 2014).

## 6 Implications for the research

The hypotheses formulated in the 5D model invite further research, testing, and refinement. The central and operational hypotheses, summarized in Table 1, carry numerous implications for scientific inquiry. This section briefly outlines several major directions for future studies.

### 6.1 Mixed-methods research on a transdiagnostic approach to the etiology of (mental) health disorders

Such research would identify whether there are typical emotional dispositions underlying specific mental and psychosomatic disorders, and possibly extend to general health disorders, given the 5D model’s holistic view and the central role of emotion in homeostasis. The ideal design would integrate *personal history* as causal context (e.g., life script, attachment patterns, anamnesis), *emotional dispositions* (levels of emotional awareness, information-processing aspects of core emotions, “initial mistake” detection, and discrete emotional profiles by frequency, duration, intensity, and regulation), *levels of consciousness* (inferred *via* validated measures of basic values and conflict management), *physiological homeostasis* (e.g., HRV or similar indicators), and *(mental) health outcomes* (self-report tools or diagnosis-based groups). Data would be triangulated across subjective (self-report), observational (symptoms, diagnosis, behavior), and objective (psychophysiological) measures. While such a design is best suited for smaller samples and would require the development of new instruments, it offers rich potential and will be explored further in future studies.

### 6.2 Correlational studies

Table 1 outlines numerous potential correlations that could be tested on large samples. Many existing studies have examined combinations of the proposed variables, with findings that align with the continuum suggested in the 5D model. Notably, research has explored correlations and network ties among personal values, emotional intelligence, and mental health (Jacobs and Wollny, 2022); conflict management and emotional intelligence (Rahim et al., 2002); affect, attachment, and psychopathology (Fuchshuber et al., 2024); and attachment, primary emotions, and spirituality (Freund et al., 2024). Studies of this kind could further enrich the evidence base by incorporating person-centered analyses. Beyond examining variable correlations, they could employ latent profile analysis to investigate whether distinct patterns emerge that are consistent with the 5D model’s proposed levels of consciousness.

### 6.3 Experimental studies

Negative attitudes toward subjective experience took root in psychiatry and allied fields decades ago, when there were few avenues for scientifically studying it. Taschereau-Dumouchel et al. (2022) responded to this legacy by calling to “put the mental back” into mental disorder research, emphasizing the need to prioritize subjective emotional experience alongside physiological and behavioral measures. For the development of emotional literacy, unconscious emotions are particularly relevant—making the contributions of Wiens (2006), who highlighted persistent difficulties in reliably assessing conscious emotional awareness, and Frumento et al. (2022), who introduced the concept of *emotionally-subliminal stimuli*, especially significant. These stimuli—consciously perceived yet failing to evoke the expected emotional response—refine traditional definitions of subliminality and offer therapeutic potential. This line of research is fully aligned with the 5D model and could be strengthened by experimental designs that control for physiological homeostasis and other outlined aspects of emotional dispositions.

### 6.4 Cross-cultural studies

The 5D model outlines a universal developmental continuum across cultures and histories, while valuing the richness of indigenous psychologies and traditions. It recognizes differences in societal development and the power dynamics that shape whose voices and knowledge are privileged. For cross-cultural studies, it promotes the *transformative research paradigm* (Mertens, 2007), which rejects cultural relativism and examines how power operates in the construction of knowledge. Grounded in social justice, this paradigm integrates feminist, critical race, postcolonial, indigenous, disability, and LGBTQ perspectives to challenge ethnocentric assumptions. Through culturally responsive, collaborative research, it centers the values and lived experiences of marginalized communities, addresses inequities in researcher–participant relationships, and generates knowledge aimed at equitable, inclusive social change by situating and describing the context—particularly the power relations—that shape the production and reception of knowledge.

### 6.5 Multidisciplinary studies

The 5D model views all functions of an organism as part of a coordinated feedback loop aimed at growth, requiring insights from multiple disciplines. Constructive Realism (Wallner and Jandl, 2006) supports such integration, distinguishing between reality as the sum of scientifically constructed “microworlds,” *Wirklichkeit* as the actual world that exists but remains inaccessible, and the life-world of everyday experience. In this view, understanding deepens through *strangification*—examining one microworld from the standpoint of another to gain fresh perspectives. This approach can bridge ancient wisdom and emerging biophysical models, such as linking “gut feelings” with gut–brain communication (Mayer, 2011) or connecting the pineal gland—traditionally the “third eye”—with neuroscientific findings on its role in dreams, meditation, and altered states (Bellec, 2024). Similarly, phrases like “fear in the bones” could inspire research into early trauma, chronic freeze responses, and gut-axis disruptions, offering new healing pathways. The broader treatment and research implications of the 5D model are significant but beyond the scope of this article.

## 7 Discussion

**Figure 6:**
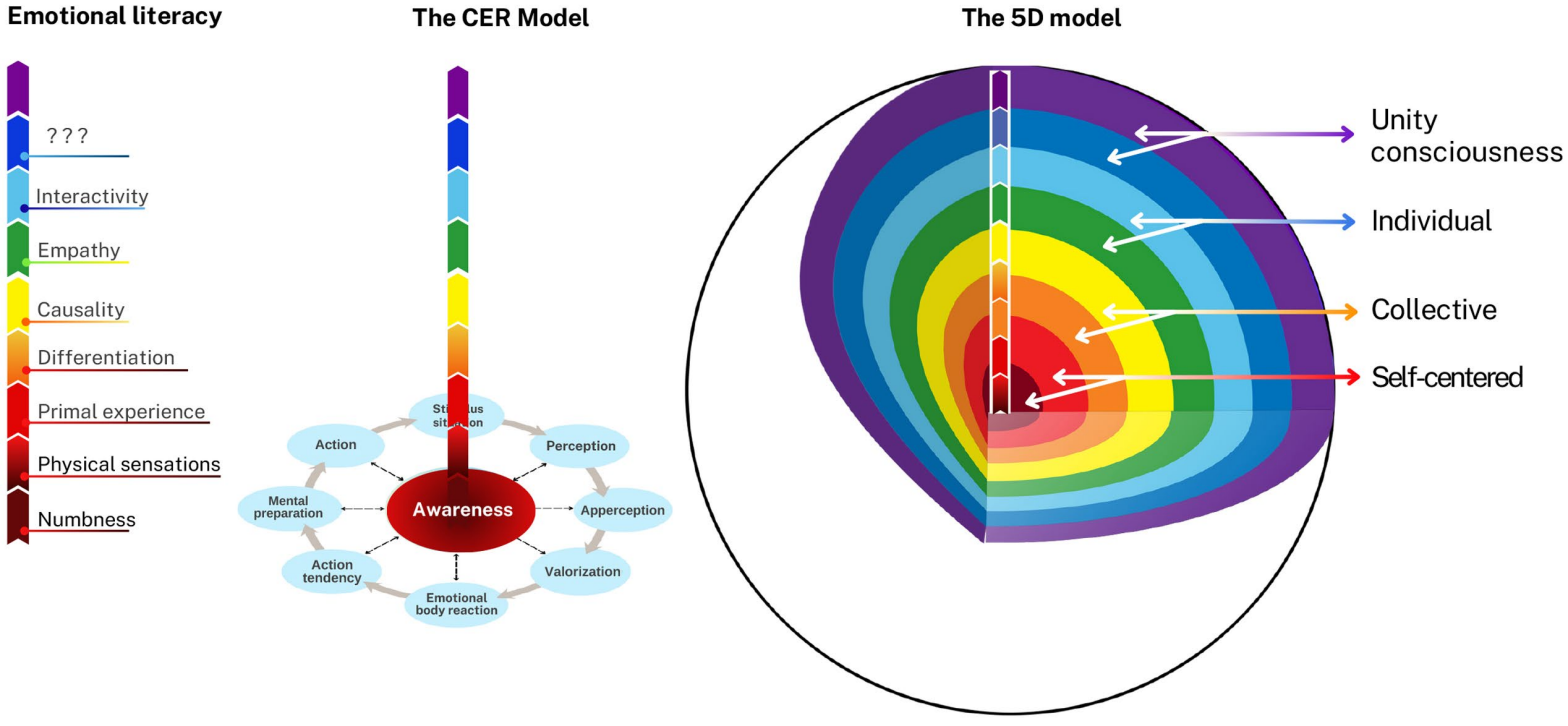
Evolution and integration of the emotional literacy and the CER model into the 5D model. Steiner (2003) developed the emotional literacy dimension, represented on the upward-pointing scale, and intentionally left open the possibility for higher expressions. Milivojevic (2000) built the CER model as a feedback loop, placing emotional awareness and literacy at its center to present a psychology and psychopathology framework of emotions. The 5D model extends this emotional literacy axis into the spiritual and ecological domains, proposing four levels of consciousness and two higher stages of emotional literacy—emotional creativity and transformative power—to capture the highest expressions of unity consciousness. In this model, levels of consciousness are linked to emotional literacy through CER, symbolized by rainbow-like colors representing both frequency and the functional unityof body and mind, underscoring that all functions of an organism operate in unison within a feedback loop oriented toward growth and adaptation.

The integration of emotional literacy with the CER model and its subsequent operationalization resulted in the development of a novel 5D framework. Figure 6 illustrates the evolution and synthesis of its components into the bio-psycho-social-spiritual-ecological (5D) model of human experience. It unites major psychological functions across the lifespan—at intrapersonal, interpersonal, and societal levels—within both an energetic (5D) and material (3D) framework. The model identifies four levels of consciousness, shaped by nature–nurture interplay, and proposes that they are directly proportional to emotional literacy in the physical realm, operationalized through the CER mechanism. Drawing on analogies from physics, such as relativity and wave–particle duality, it links physiological, motivational, and cognitive processes into a single feedback system. Suboptimal conditions can halt development at lower levels, while higher levels manifest as EC and transformative power. These constructs are anchored in prior empirical findings and positioned along a continuum of physiological homeostasis, motivational needs, mental health outcomes, and observable psychological functions. In its essence, the 5D model reflects the interconnectedness and wholeness of human experience—an idea beautifully captured by the 13th-century Persian poet Rumi: *“You are not a drop in the ocean. You are the entire ocean in a drop”* (Jalal ad-Din Muhammad ar-Rumi, n.d.).

Taken together, the developed 5D model offers a comprehensive theoretical framework for emotion psychology and psychopathology, addressing all five questions posed by Reisenzein (2015). It further suggests that emotional development is inseparable from moral development, which in turn equates to spiritual development—each grounded in the laws of nature and the universe and reflecting a universal progression along a developmental continuum from infancy to maturity, across time and space, evolving from a state of being rooted in fear to one grounded in love. The model aligns with emerging biofield science (Rubik et al., 2015) and calls for empirical testing of its hypotheses and methodology, particularly within a transdiagnostic approach to the etiology of health disorders. It also emphasizes the value of multidisciplinary research, where scientifically validated methods—supported by converging subjective, objective, and observable measures—could ultimately enable objective assessment of consciousness, mental health, and wellbeing through indicators such as HRV and the heart’s electromagnetic field (McCraty et al., 2009).

The ecological domain reveals how adverse conditions—war, poverty, disasters, political oppression, subtle power abuse, and cultural norms that stifle emotional expression—can arrest psychological growth, especially in early life. Unstable caregiving, unresolved trauma, and limited emotional literacy weaken secure attachment, locking individuals into cycles of fear and disconnection. From these lowest developmental rungs often emerge those who hunger for absolute ‘power over,’ leaving devastation in their wake, as history shows in the legacies of Hitler, Stalin, and Mao (with very similar family histories; Goldner-Vukov and Moore, 2010) — echoed today in certain leaders whose personal wounds shape global ruin. In contrast, figures such as Satir, Maslow, Kohlberg, and Martin Luther King Jr. reached toward self-transcendence, grounding their visions in love, unity, and justice, and leaving legacies that expand the horizon of human possibility. King’s belief in love as “the force which all of the great religions have seen as the supreme unifying principle of life” (Martin Luther King, Jr. Quote, n.d.) resonates with the timeless insight that only those grounded in integrity, virtue, and moral wisdom should guide society—a truth Plato crystallized in his call for philosopher kings, leaders whose inner development equips them to safeguard the common good.

The ultimate vision of the 5D model is to translate ancient universal spiritual truths into a unified scientific language for our time. By aligning with our true sense of purpose, joining our inner transformative power with that of others, and speaking and acting Truth to Power, we can work together to replace “power over” with “power to the people.” As democracies—long regarded as the best form of governance—have shown themselves vulnerable to corruption, one corrective could be to develop and require objective, validated measures of integrity, as prerequisites for candidacy in public office. With such foundations in place, conversations can shift toward how best to care for the *Anima Mundi* and to nurture a desperately needed global perspective of healing—one that embraces both traditional wisdom and modern psychotherapies (Pritz, 2002). And in this shared journey, every new hue that appears in the rainbow of human growth is a victory for the collective, advancing the evolution of consciousness and the conscious evolution itself.

## Data Availability

The original contributions presented in the study are included in the article/Supplementary material, further inquiries can be directed to the corresponding author.

## Footnotes

1 In the 19th century, the nature of light was known as a wave-like nature that can be dispersed through a prism, showing rainbow colors. In 1905, Albert EinACstein demonstrated a so-called “photoelectric” effect, which proved that light could strike electrons—called “photons.” Twenty years later, Louis de Broglie realized that, far from being a problem, light (and any material particle) behaves like both a wave and a particle that shares the same frequency. (Wave-particle duality, 2024).
