# Supplementary material for: Emotional literacy and levels of consciousness in bio-psycho-social-spiritual-ecological (5D) model of human experience
Source: Front Psychol. 2026 Jan 22;17:1520959. doi: 10.3389/fpsyg.2026.1520959 (PMC12872479; doi:10.3389/fpsyg.2026.1520959)
Supplement: Supplementary file 1 [file Data_Sheet_1.pdf]

Supplementary Material 1

Supplementary Material for the article: "Emotional literacy and levels of consciousness in bio-psycho-social-spiritual-ecological (5D) model of human experience"

Author: Ivana Dragic

Description of the visible iceberg layers and spiritual practices in the 5D Model

This supplementary document provides an expanded explanation of the layers above the waterline in the Personal Iceberg metaphor of the 5D model at the intrapersonal, interpersonal, and societal levels, with relevant theoretical foundations indicated in Figure 4 and Table 1. It further expands on the layer well below the waterline related to love, compassion, and spiritual practices.

1.1 Self-awareness, personality, and public self

Self-awareness. The term *self-awareness* refers to the capacity to focus attention inward and to become a reflective observer processing self-information (as a higher level of consciousness), whereas *consciousness* refers to focusing attention outward on the environment and processing incoming external stimuli (Morin, 2006). In the state of self-awareness, our attention may be directed either toward *private* aspects of the self—such as emotions, values, and goals—or *public* aspects, like physical appearance and behavior. Focusing on private aspects represents a more advanced form of self-awareness, as it involves processing more abstract and less observable information. At the highest level of consciousness is *meta-self-awareness*—being aware that one is aware. Morin (2006) further distinguishes between the *quantity* of self-awareness (how often one reflects on the self) and its *quality* (the accuracy of that self-perception), noting that these dimensions do not necessarily correlate. Self-examination can take the form of healthy *self-reflection*, characterized by curiosity and introspection, or maladaptive *self-rumination*, marked by anxiety and obsessive self-questioning. These variations contribute to different levels of self-awareness and consciousness, particularly in how accurately internal and external information is processed. This concept is central to the 5D model, which uses the metaphor of a *Personal Iceberg* to illustrate the depth of conscious interpretation—highlighting how much lies beneath the surface in the preconscious (latent but accessible) and unconscious mind, a framework originally introduced by Sigmund Freud (1962) and expanded by Carl Gustav Jung (1939).

Personality. Among the many theories of personality, those most relevant to levels of consciousness are the pioneering works of Freud and Jung. Freud (1989) proposed a tripartite structure of personality: the *id* (an unconscious reservoir of instinctual drives and psychic energy governed by the pleasure principle), the *ego* (the rational mediator that emerges from the id through interactions with the external world, operating on the reality principle), and the *superego* (the internalized moral standards of parents and society). The ego exists across conscious, preconscious, and unconscious levels (as does the superego) and is tasked with reconciling the conflicting demands of the id, superego, and the external world. According to Freud, the dynamics established among these

components during childhood shape the foundation of an individual's personality. Jung offered a different perspective. He did not see the id as the primary source of psychic energy, but rather as just one aspect of it. Jung (1968) proposed that each person is born with an innate sense of *wholeness*—the *Self*—which serves as the psychic nucleus of the total personality, encompassing consciousness, the unconscious, and the ego. As development progresses, ego-consciousness emerges from this original unity. In Jung's view, only after the ego has matured and been successfully integrated into the external world (typically not before midlife) can an individual rediscover the Self through the transformative process of *individuation*.

Public self. The *public self* represents how we present ourselves to others—and how we wish to be seen. Jung (1967, par. 305) referred to this facet of personality as the *persona*, describing it as "a kind of mask designed on the one hand to make a definite impression upon others, and, on the other, to conceal the true nature of the individual". The persona is a complex interface between the individual and the social environment, continuously shaped by societal expectations and developmental stages. As we grow and adapt, we develop different public selves to meet the demands of varying social contexts. When self-awareness is focused exclusively on public aspects—such as appearance, behavior, social status, or others' opinions—it reflects a lower level of self-awareness compared to when it is directed toward private, internal experiences. This is because public aspects rely on readily perceptible information, whereas private aspects require more abstract reflection (Morin, 2006).

1.2 Cognition, communication styles, and behavior

Cognition. Cognition refers to the mental processes involved in acquiring, storing, and using knowledge. Piaget's "genetic epistemology" (the study of the nature and origins of knowledge) is a widely accepted theory of cognitive development, which emphasizes that children actively *construct knowledge* through interaction with the world. According to Piaget (1964), schemas are fundamental building blocks of cognitive development that are constantly being created, modified, and reorganized by balancing assimilation (fitting new information into already existing schemas) and accommodation (adjusting existing schemas according to novel information). As the brain develops, children move through these four stages: *the sensorimotor stage* (birth to 2 years), characterized by exploration through senses and movement; *the preoperational stage* (ages 2 to 7 years), characterized by symbolic but illogical thinking; *the concrete operational stage* (ages 7 to 11 years), characterized by logical thinking about concrete events; and *the formal operational stage*, characterized by the logical use of symbols related to abstract concepts. However, the fully developed formal operational stage is not universally achieved (Kuhn et al., 1979). The described stages are first-order cognitive skills that enable one to know about the world. Further development implies *second-order metacognitive skills* that entail knowing about one's own (and others') knowing, which are essential for *critical thinking* - "the competencies that enable people to participate fully as citizens in a democracy" (Kuhn, 1999, p. 16). However, meta-knowing competencies, in contrast to most of the competencies in developmental psychology, remain incompletely developed (Kuhn, 1999). The highest form of cognition can be considered *Being cognition* - a contemplative way of knowing oriented toward the highest goods, as described by Maslow (1971). Being cognition involves a dialectical blending of experiential awareness of the interrelatedness between the perceiver and the whole universe with complete absorption in a single object, phenomenon, person, or work, such that the rest of the universe, including the perceiver, appears to disappear (Maslow, 1971).

Communication. At the interpersonal level, cognition is expressed through communication, encompassing verbal and nonverbal forms as well as various artifacts of cognitive functioning. In the 5D model, the most relevant concept for communication is *assertiveness,* understood as a prerequisite for self-actualization. Assertiveness refers to the capacity to recognize and protect one's own human rights in a respectful and democratic manner, without compromising the rights of others (Peneva and Mavrodiev, 2013). Lazarus (1973) described four fundamental abilities of assertive communication: openly expressing desires and needs, saying "no", openly expressing feelings, and establishing interpersonal connections. Neglecting other people's rights gives rise to *aggressive communication*, neglecting one's own rights leads to *passive communication*, and neglecting the rights of both parties while avoiding direct communication leads to *passive-aggressive* style. Highly assertive communication, combined with a cooperative orientation, reflects *collaborative* skills that are associated with improved psychological health, social competence, achievement, and more fulfilling relationships (Laal and Ghodsi, 2012).

Behavior. Within the broad domain of observable behavior, Eric Berne's transactional analysis provides the most relevant framework for the 5D model, as articulated in *Games People Play* (1964). Berne conceptualized behavior in terms of *how individuals structure time*, emphasizing that behavior manifests as concrete action within a specific situational and temporal context. In social interaction, time may be structured through rituals (e.g., culturally learned manners), pastimes (e.g., topical conversations), games, intimacy, and activity, which may take the form of combinations of these modes. *Games* are defined as series of complementary transactions driven by concealed motivations that produce emotional payoffs and culminate in predictable outcomes. Every game is fundamentally dishonest, and its outcome has a dramatic quality. Stephen Karpman (1968) further explicated the underlying dynamic of games through the Drama Triangle concept. Only three roles are required to depict emotional reversals and sudden dramatic shifts: Persecutor, Victim, and Rescuer. *Persecutors* are characterized by aggression, hypercriticism, and perceived superiority and entitlement, frequently oppressing and blaming others for their difficulties. *Victims* experience themselves as powerless and helpless, struggle with decision-making and problem-solving, and commonly exhibit fear, anxiety, and depressive affect. *Rescuers*, driven by people-pleasing tendencies, intervene excessively in others' lives, assuming they know what is best and deriving self-importance and emotional reward from attempts to improve, change, or control others' behavior (Lac and Donaldson, 2020). According to Berne (1964), becoming aware of interpersonal games and their motivational underpinnings enables individuals to disrupt maladaptive patterns. By cultivating authentic communication and emotional honesty, individuals can move toward *intimacy* and develop meaningful relationships characterized by mutuality and balance.

1.3 Values, attachment, and social identity

Values. Shalom Schwartz's Theory of Basic Human Values (Schwartz, 2012) is among the most influential and extensively validated cross-cultural theories of values. The theory conceptualizes values as fundamental motivators of behaviors and attitudes and identifies ten basic personal values that are widely recognized across cultures. These values are organized within a circular structure that captures systematic patterns of compatibility and conflict among them. The ten motivational domains are integrated into four third-order value categories: 1) *self-transcendence* (universalism and benevolence), prioritizing concern for the welfare of society and nature over personal interests, and emphasizing acceptance of others as equals; 2) *conservation* (security, conformity, tradition), prioritizing stability, order, and self-restraint; 3) *self-enhancement* (achievement, power), prioritizing personal success and dominance over others; and 4) *openness to change* (hedonism, stimulation, self-direction), prioritizing independence of thought and action and autonomous regulation of behavior.

Cross-cultural research demonstrates a strong consensus regarding the relative importance of the ten values across societies: "benevolence, universalism, and self-direction values appear at the top of the hierarchy and power, tradition, and stimulation values appear at the bottom. This implies that the aspects of human nature and of social functioning that shape individual value priorities are widely shared across cultures" (Schwartz, 2012, p. 17).

Attachment. In the interpersonal domain, values and interpersonal attraction are expressed through personal relationships, within which the concept of attachment plays a central role. Influenced by ethology, Bowlby (1969) conceptualized attachment as an innate emotional bond between infants and familiar caregivers that develops during a critical early period – most prominently within the first two years of life – as an evolutionary adaptation supporting infant survival. He proposed that early relationships with primary caregivers give rise to internal "working models", which shape generalized expectations about the self, others and the world. Ainsworth et. al., (1978/2015) delineated three primary attachment styles emerging in response to caregiving behaviors: *secure attachment,* associated with sensitive and responsive caregiving that provides a secure base for exploration; *ambivalent/anxious attachment*, associated with inconsistent caregiving that fosters anxiety regarding caregiver availability and limits exploratory behavior; and *avoidant attachment*, associated with rejection of proximity and chronic neglect, leading to defensive self-reliance and suppression of attachment-related behaviors and emotions. A fourth attachment style, *disorganized,* was later identified by Main and Solomon (1986) and describes responses to abusive or frightening caregivers, in which infants experience fear toward the same individuals on whom they depend for protection, resulting in disorganizing conflict between impulses to flee and to seek proximity.

Across development, these early attachment patterns may either persist or transform through reflective self-work, emotionally safe relationships, and psychological growth. Psychological maturity introduces the possibility of *non-attachment,* understood not as disengagement but as a state of equanimity, inner freedom, and compassionate presence. While early attachment is essential for survival, non-attachment represents a later developmental capacity characterized by relational flexibility, grounded in love rather than need and connection rather than control. Empirical research indicates that non-attachment, as assessed by the Nonattachment Scale (Sahdra et al., 2010, 2015), is associated with enhanced emotional well-being, mindfulness, empathy, prosocial behavior, and reduced rumination, thereby supporting healthier relational dynamics and greater inner peace in adulthood.

Social identity. Social Identity Theory, formulated by Tajfel and Turner (1979), posits that individuals derive a substantial portion of their self-concept from membership in social groups, including categories such as age, social class, sports teams, musical preferences, and political affiliation. Social identity refers to an individual's sense of self derived from group memberships, which serve as important sources of belonging, pride, self-esteem, and purpose. The development of social identity involves a sequence of interrelated processes, beginning with *social categorization*, through which individuals classify themselves and others into social groups (e.g., race, gender, nationality, or religion) to simplify the social environment. This is followed by *social identification*, whereby individuals internalize the norms, values, and behaviors of groups they perceive themselves to belong to, and *social comparison*, in which in-groups are evaluated relative to out-groups, often leading to in-group favoritism and the development of stereotypes and prejudices. These processes give rise to *in-group* ("us") and *out-group* ("them") distinctions, fostering perceptions of the in-group as superior and the out-group as inferior or threatening, thereby increasing the likelihood of rivalry,

competition, and hostility. The motivation for *positive distinctiveness*, driven by the need for self-esteem, can further exacerbate prejudice and discrimination and, in extreme manifestations, give rise to racism and genocide. By identifying the psychological mechanisms and social conditions that underlie intergroup dynamics—particularly those related to prejudice, bias, and discrimination—*research-informed interventions can be designed to foster intergroup tolerance, cooperation, inclusion, and peacebuilding.*

1.4 Love, compassion, and spiritual practices

At the interpersonal level, *loving* relationships among romantic partners, families, and friends are widely recognized as a universal phenomenon, although their expression and importance within intimate relationships vary across cultures and historical periods (Rokach, 2024). Awareness of universal human yearnings and the interconnectedness of all human beings with a universal life force gives rise to *compassion*, which "consist[s] of five elements: recognizing suffering, understanding the universality of human suffering, feeling for the person suffering, tolerating uncomfortable feelings, and motivation to act/acting to alleviate suffering" (Strauss et al., 2016, p. 15). Compassion is not limited to attachment-based bonds with significant others but extends to unfamiliar individuals and humanity at large. Across major world religions, love and compassion are emphasized as central ethical principles (Strauss et al., 2016).

Spiritual practices are activities that can induce and cultivate spiritual development, if practiced regularly. They may take various forms and include combinations of practices such as self-reflection, meditation, solitude, intimacy, yoga, dancing, singing, drawing, chanting, praying, and fasting. *Meditation,* in particular, encompasses a range of styles and techniques found across cultural and historical contexts, originating from contemplative traditions including Buddhist, Christian, Hindu, Sufi, yogic, shamanic, and transcendental practices. Growing interest in meditation within cognitive neuroscience has led to cross-cultural classification of meditation styles and to accumulating evidence of its effects, which include enhanced attention and sensory processing, predominantly positive emotional states, increased cognitive flexibility and creativity, and, in some cases with greater expertise, experiences described as "ego dissolution" (Millière et al., 2018). Experiences of ego dissolution - an altered state of consciousness also referred to as "self-loss", "non-dual awareness", or "pure consciousness" - may take different forms, and preliminary evidence suggests enduring effects on cognitive functioning, personality traits (such as selflessness) and prosocial behavior (Millière et al., 2018). In Jungian terms (Jung, 1968), ego dissolution represents a critical aspect of the individuation process, enabling contact with the collective unconscious and the realization of the Self.

References:

Ainsworth, M. D. S., Blehar, M. C., Waters, E., and Wall, S. N. (1978). *Patterns of attachment: A psychological study of the strange situation* (Classic edition). New York, NY; London: Routledge/Taylor & Francis Group. doi:10.4324/9780203758045

Berne, E. (1964). *Games people play: The psychology of human relationships*. New York, NY: Grove Press.

Bowlby, J. (1969). *Attachment and loss*, Vol. 1: Attachment. New York, NY: Basic Books.

Freud, S. (1962). *Fragment of an analysis of a case of hysteria* (1905 [1901]). London: Hogarth Press.

Freud, S. (1989). *The ego and the id* (1923). New York, NY: W. W. Norton.

Jung, C. G. (1939). *The integration of the personality*. New York, NY: Farrar & Rinehart.

Jung, C. G. (1967). *Collected works of C. G. Jung*, Vol. 7. Princeton, NJ: Princeton University Press.

Jung, C. G. (1968). *Man and his symbols* (Reissue edition). New York, NY: Dell.

Karpman, S. B. (1968). Fairy tales and script drama analysis. *Transactional Analysis Bulletin*, 7, 39–43.

Kuhn, D. (1999). A developmental model of critical thinking. *Educational Researcher*, 28(2), 16–25. doi:10.3102/0013189X028002016

Kuhn, D., Ho, V., and Adams, C. (1979). Formal reasoning among pre- and late adolescents. *Child Development*, 50, 1128–1135. doi:10.2307/1129340

Laal, M., and Ghodsi, S. M. (2012). Benefits of collaborative learning. *Procedia – Social and Behavioral Sciences*, 31, 486–490. doi:10.1016/j.sbspro.2011.12.091

Lac, A., and Donaldson, C. (2020). Development and validation of the Drama Triangle Scale: Are you a victim, rescuer, or persecutor? *Journal of Interpersonal Violence*, 37, NP992–NP1017. doi:10.1177/0886260520957696

Lazarus, A. A. (1973). On assertive behavior: A brief note. *Behavior Therapy*, 4, 697–699. doi:10.1016/S0005-7894(73)80161-3

Main, M., and Solomon, J. (1986). Discovery of an insecure–disorganized/disoriented attachment pattern. In T. B. Brazelton and M. W. Yogman (Eds.), *Affective development in infancy* (pp. 95–124). Norwood, NJ: Ablex Publishing Corporation.

Maslow, A. H. (1971). *The farther reaches of human nature*. New York, NY: Arkana/Penguin Books.

Millière, R., Carhart-Harris, R. L., Roseman, L., Trautwein, F.-M., and Berkovich-Ohana, A. (2018). Psychedelics, meditation, and self-consciousness. *Frontiers in Psychology*, 9. doi:10.3389/fpsyg.2018.01475

Morin, A. (2006). Levels of consciousness and self-awareness: A comparison and integration of various neurocognitive views. *Consciousness and Cognition*, 15, 358–371. doi:10.1016/j.concog.2005.09.006

Peneva, I., and Mavrodiev, S. (2013). A historical approach to assertiveness. *Psychological Thought*, 6, 3–26. doi:10.5964/psyct.v6i1.14

Piaget, J. (1964). Part I: Cognitive development in children: Piaget development and learning. *Journal of Research in Science Teaching*, 2, 176–186. doi:10.1002/tea.3660020306

Rokach, A. (2024). The meanings of love: An introduction. *Journal of Psychology*, 158, 1–4. doi:10.1080/00223980.2024.2307284

Sahdra, B. K., Ciarrochi, J., Parker, P. D., Marshall, S., and Heaven, P. (2015). Empathy and nonattachment independently predict peer nominations of prosocial behavior of adolescents. *Frontiers in Psychology*, 6, 263. doi:10.3389/fpsyg.2015.00263

Sahdra, B. K., Shaver, P. R., and Brown, K. W. (2010). A scale to measure nonattachment: A Buddhist complement to Western research on attachment and adaptive functioning. *Journal of Personality Assessment*, 92, 116–127. doi:10.1080/00223890903425960

Schwartz, S. H. (2012). An overview of the Schwartz theory of basic values. *Online Readings in Psychology and Culture*, 2(1). doi:10.9707/2307-0919.1116

Strauss, C., Lever Taylor, B., Gu, J., Kuyken, W., Baer, R., Jones, F., et al. (2016). What is compassion and how can we measure it? A review of definitions and measures. *Clinical Psychology Review*, 47, 15–27. doi:10.1016/j.cpr.2016.05.004

Tajfel, H., and Turner, J. C. (1979). An integrative theory of inter-group conflict. In W. G. Austin and S. Worchel (Eds.), *The social psychology of inter-group relations* (pp. 33–47). Monterey, CA: Brooks/Cole.
